# Interstitial macrophage phenotypes in *Schistosoma*-induced pulmonary hypertension

**DOI:** 10.3389/fimmu.2024.1372957

**Published:** 2024-05-08

**Authors:** Rahul Kumar, Sushil Kumar, Claudia Mickael, Dara Fonseca Balladares, Kevin Nolan, Michael H. Lee, Linda Sanders, Julia Nilsson, Ari B. Molofsky, Rubin M. Tuder, Kurt R. Stenmark, Brian B. Graham

**Affiliations:** ^1^Department of Medicine, University of California, San Francisco, San Francisco, CA, United States; ^2^Lung Biology Center, Zuckerberg San Francisco General Hospital, San Francisco, CA, United States; ^3^Department of Pediatrics and Cardiovascular Pulmonary Research Laboratory, University of Colorado Anschutz Medical Campus, Aurora, CO, United States; ^4^Division of Pulmonary Sciences and Critical Care Medicine, Department of Medicine, University of Colorado Anschutz Medical Campus, Aurora, CO, United States; ^5^Department of Laboratory Medicine, University of California San Francisco, San Francisco, CA, United States

**Keywords:** macrophages, inflammation, schistosomiasis, pulmonary hypertension, type 2 inflammation

## Abstract

**Background:**

Schistosomiasis is a common cause of pulmonary hypertension (PH) worldwide. Type 2 inflammation contributes to the development of Schistosoma-induced PH. Specifically, interstitial macrophages (IMs) derived from monocytes play a pivotal role by producing thrombospondin-1 (TSP-1), which in turn activates TGF-β, thereby driving the pathology of PH. Resident and recruited IM subpopulations have recently been identified. We hypothesized that in Schistosoma-PH, one IM subpopulation expresses monocyte recruitment factors, whereas recruited monocytes become a separate IM subpopulation that expresses TSP-1.

**Methods:**

Mice were intraperitoneally sensitized and then intravenously challenged with S. mansoni eggs. Flow cytometry on lungs and blood was performed on wildtype and reporter mice to identify IM subpopulations and protein expression. Single-cell RNA sequencing (scRNAseq) was performed on flow-sorted IMs from unexposed and at day 1, 3 and 7 following Schistosoma exposure to complement flow cytometry based IM characterization and identify gene expression.

**Results:**

Flow cytometry and scRNAseq both identified 3 IM subpopulations, characterized by CCR2, MHCII, and FOLR2 expression. Following *Schistosoma* exposure, the CCR2^+^ IM subpopulation expanded, suggestive of circulating monocyte recruitment. *Schistosoma* exposure caused increased monocyte-recruitment ligand CCL2 expression in the resident FOLR2^+^ IM subpopulation. In contrast, the vascular pathology-driving protein TSP-1 was greatest in the CCR2^+^ IM subpopulation.

**Conclusion:**

*Schistosoma*-induced PH involves crosstalk between IM subpopulations, with increased expression of monocyte recruitment ligands by resident FOLR2^+^ IMs, and the recruitment of CCR2^+^ IMs which express TSP-1 that activates TGF-β and causes PH.

## 1 Introduction

Pulmonary vascular homeostasis is essential for maintaining respiratory health but is dramatically perturbed during disease states such as pulmonary hypertension (PH). PH can be incited by infection with the parasite Schistosoma, causing schistosomiasis, a significant global health challenge (1). The parasite’s ability to trigger Type 2 immune responses, characterized by cytokines including IL-4 and IL-13 (2), is a crucial aspect of the pathogenesis that leads to pulmonary vascular remodeling—the pathological hallmark of PH.

Interstitial macrophages (IMs), derived from circulating monocytes, have been implicated in this process (3, 4). Their role is multifaceted, involving not just the orchestration of immune responses but also direct engagement in the vascular remodeling process through the expression of thrombospondin-1 (TSP-1) (3). TSP-1 is a matricellular protein that, by activating the latent TGF-β complex, contributes to the fibrotic remodeling of the pulmonary vasculature (5). The intricacies of this process are underscored by potentially different roles of resident and recruited IM subpopulations, each with distinct phenotypic markers and unique contributions to disease progression.

Our research is guided by a central hypothesis: that Type 2 immunity-driven IMs are key producers of monocyte recruitment ligands following Schistosoma exposure, while another subset is responsible for TSP-1 expression, with both contributing to the pathogenesis of PH. The novelty of our approach lies in the integration of flow cytometry with complementary single-cell RNA sequencing (scRNAseq) to dissect the phenotypic nuances of these IM subpopulations. Through this dual-modality analysis, we aim to unravel the molecular dialogue between these cells and their role in promoting vascular pathology.

By identifying the specific phenotypes of these IM subpopulations, we expect to uncover new molecular mechanisms underlying Schistosoma-induced PH. This could provide a paradigm shift in our understanding of PH and propel the development of targeted therapies. Moreover, this research has the potential to shed light on broader mechanisms involving detailed cartography of immune cell function within the pulmonary vascular niche, also applicable to other forms of PH, thus broadening its impact.

## 2 Methods

### 2.1 Animals

Wildtype (C57BL6/J; stock # 000664), *Ccr2^RFP^Cx3cr1^GFP^* dual-reporter mice (stock # 032127), *Ccl2^RFP^* floxed (stock # 016849), and *Cx3cr1^GFP^* homozygote reporter mice (stock # 005582) were procured from Jackson Laboratories (Bar Harbor, ME). Sex-matched *Cx3cr1^GFP/+^* reporter littermates were bred by crossing *Cx3cr1^GFP^* homozygote reporter mice with wildtype for flow sorting immune cells. The mice were housed at the University of California, San Francisco (UCSF) or the University of Colorado Anschutz Medical Campus (CUAMC) specific pathogen-free animal facilities. All animal experiments were conducted in accordance with protocols approved by the UCSF and CUAMC Institutional Animal Care and Use Committees.

### 2.2 *Schistosoma* exposure

Mice infected with *Schistosoma mansoni* sourced from the Biomedical Research Institute, NIH, underwent euthanasia through a slow-rate CO2 infusion, ensuring gradual entry into the chamber to achieve unconsciousness and complete narcotization before death as per our IACUC approved protocol. Subsequently, eggs were collected from the liver. Experimental mice were exposed to *Schistosoma* eggs following our established protocol (3, 6). In brief, the mice were sensitized intraperitoneally (IP) with *Schistosoma* eggs, followed 2 weeks later by intravenous (IV) challenge with *Schistosoma* eggs. *Schistosoma*-unexposed mice served as controls.

### 2.3 Lung digestion procedure

Lung tissues were collected from mice following IV egg challenge at the timepoints indicated. Mice were anesthetized intraperitoneally (IP) using a mixture of ketamine (100mg/kg)/xylazine (40mg/kg). To differentiate between interstitial and circulating cells, the mice were retro-orbitally injected with an AF700 labeled anti-CD45 antibody (1µg/mouse) 5 minutes prior to euthanasia to label intravascular leukocytes. Subsequently, the lungs were perfused via the right ventricle with PBS. The PBS-flushed lungs were then collected and individually subjected to digestion following an established protocol (7). In brief, the perfused lung tissues were enzymatically digested using liberase (Roche, Germany) dissolved in RPMI medium (Mediatech, Corning, NY) at a concentration of 1mg/ml, at 37°C for 30 minutes. The tissue was further mechanically disrupted by passage through 16ga and 18ga needles five times each. Subsequently, the cells were filtered through a 100μm cell strainer (Fisher Scientific) and then centrifuged for 5 minutes at 1200 rpm. Red blood cells (RBCs) were lysed using 1ml of ACK lysis buffer (Gibco). Following RBC lysis, the cells were resuspended and washed in RPMI to neutralize the lysis buffer. The resulting single-cell suspensions were filtered and collected into flow wash buffer (5% BSA in PBS with EDTA) for subsequent staining.

### 2.4 Flow cytometry

The digested single cell suspension was pre-incubated with a CD16/CD32 antibody (clone 93; eBioscience) for 20 minutes to block non-specific Fcγ receptor-mediated antibody binding. The cells were then stained with fluorochrome conjugated antibodies at 4°C for 30 minutes. For nuclear Ki67 and intracellular TSP-1 staining, following extracellular staining the cells were fixed and permeabilized first using Fixation/Permeabilization buffer (eBioscience, Cat# 00-5523-00), and then stained using the appropriate antibodies. The details of the clone and the concentration of the extracellular and intracellular antibodies used are presented in **Supplementary Table 1**. Cell viability was assessed using LIVE/DEAD Fixable Viability Dye eFluor™ 450 violet stain (eBioscience, Cat# 65-0863-14) and the cell suspensions were analyzed with an LSRII (BD Biosciences) at the UCSF Core Immunology Laboratory (CIL). Results were analyzed using FlowJo software (v-10.9.0).

### 2.5 Imaging antibodies

For identification and localization of CCR2-RFP^+^ cells, CX3CR1-GFP^+^ cells and anatomical structures, lung tissues were immunostained. Primary antibodies used include anti-RFP Goat Polyclonal Antibody (1:200; Rockland Immunochemicals Inc), Chicken Polyclonal anti-GFP (1:200, Aves labs), anti-alpha smooth muscle actin (aSMA) Rabbit Polyclonal Antibody (1:200, Abcam). The following secondary antibodies were used at 1:300 dilution: Alexa Fluor 594 donkey anti-goat IgG (H+L) cross-adsorbed (Life Technologies, Thermo-Fisher), CF™ 488A donkey anti-chicken IgY (H+L), cross-adsorbed (Sigma-Aldrich) and Alexa Fluor 647 donkey anti-rabbit IgG (H+L) cross-adsorbed (Life Technologies, Thermo-Fisher).

### 2.6 Tissue preparation and imaging

Tissues were washed in 1X DPBS followed by cryoprotection in 30% sucrose for 24 hours and subsequently frozen in OCT (Thermo Scientific) on dry ice and stored at -80°C degrees. Coronal sections of 200μm were prepared from frozen tissue OCT blocks using a cryostat (Leica). Sections were washed in 1X DPBS and then permeabilized and blocked by incubating in block/perm buffer (DPBS/0.3% Triton X-100/1% BSA/1% normal mouse serum) overnight at 37°C degrees. After, samples were incubated with primary antibodies diluted in block/perm buffer at 37°C degrees for 3-4 days. Next, samples were washed in DPBS/0.3% Triton X-100/0.5% 1-thioglycerol for 1-2 days at room temperature, then incubated with secondary antibodies diluted in block/perm buffer at 37°C degrees for 3-4 days. Samples were washed in DPBS/0.3% Triton X-100/0.5% 1-thioglycerol for 1-2 day and then cleared by soaking in Ce3D clearing solution (DPBS/22% [weight/volume] N-methylacetamide/86% [weight/volume] Histodenz/0.7% Triton X-100) overnight. Samples were then mounted in fresh clearing solution and imaged. All preparations were scanned using a Nikon A1R laser scanning confocal including 405, 488, 561, and 650 laser lines for excitation and imaging with 16X/0.8 NA Plan Apo long working distance water immersion objective. Z steps were acquired every 3μm.

### 2.7 Image analysis

z-stacks images were rendered in 3D dimensions and quantitatively analyzed using Bitplane Imaris v9.6 software package (Andor Technology PLC, Belfast, N. Ireland). Individual CCR2^+^ and CX3CR1^+^ macrophages were annotated using the Imaris spots function based on the fluorescent reporter signal and using the Ortho slicer function to visualize size, morphology, and nuclear staining (DAPI). 3D reconstructions of aSMA-labeled structures were performed using Imaris surface function.

### 2.8 Macrophage flow sorting for scRNA-seq

Macrophages were flow-sorted from age and sex-matched *Cx3cr1^GFP/+^* reporter mice. The gating strategy, as illustrated in the figures, guided the precise selection of IMs and AMs. Each experimental time point involved the use of a pair of age and sex-matched mice, ensuring a balanced representation with one male and one female mouse. The flow sorting procedure was conducted employing an Astrios EQ cell sorter (Beckman Coulter Life Sciences) at the CUAMC Cancer Center Flow Cytometry Shared Resource. Following the flow sorting, the isolated cells were collected in a specialized medium (HBSS-Gibco with 2.5% FBS), subjected to centrifugation, accurately counted, and prepared for subsequent sequencing analysis. This systematic approach ensured the acquisition of high-quality data from the sorted IMs and AMs, contributing to the comprehensive understanding of their transcriptional profiles across different conditions.

### 2.9 scRNA-seq library preparation and sequencing

The sequencing process was carried out by the CUAMC Genomics Core. Sorted cells were suspended in PBS solution containing 0.05% BSA before being sequenced using a NovaSEQ 6000 sequencer (Illumina, Inc. San Diego, CA). The single‐cell 3′ Library and Gel Bead Kit V3.1 (10x Genomics, 1000268) along with the Chromium Single Cell G Chip Kit (10x Genomics, 1000120) were utilized. The cell suspension was loaded onto the Chromium Single Cell Controller (10x Genomics) to create single‐cell gel beads in the emulsion following the manufacturer’s protocol. In brief, single cells were suspended in PBS with 0.04% BSA. Approximately 10,000 cells were loaded into each channel, with an estimated recovery target of about 5,000 cells. Captured cells were lysed, and RNA was barcoded through reverse transcription in individual GEMs. Reverse transcription was performed on an S1000TM Touch Thermal Cycler (Bio Rad) at 53°C for 45 min, followed by 85°C for 5 min, and then held at 4°C. The cDNA was then amplified and assessed for quality using an Agilent 4200 system. scRNA‐seq libraries were constructed according to the manufacturer’s instructions using the Single Cell 3′ Library and Gel Bead Kit V3.1. Finally, the libraries were sequenced using an Illumina Novaseq6000 sequencer with a sequencing depth of at least 100,000 reads per cell using the paired‐end 150 bp strategy. Subsequently, FASTQ files from each sample were aligned to the mouse (Mus musculus GRCm39) reference genome using Cell Ranger v6 (10x Genomics) as per manufacturer’s guidelines.

### 2.10 scRNA-seq data analysis

Utilizing the Seurat package (v4.1.1) for scRNA-seq data (8), we generated Seurat objects. To enhance data quality, we excluded fragmented and doublet cells using DoubletFinder (v2.0.3) (9), retaining only those with over 300 distinct genes and less than 15% mitochondrial genes. Data integration from multiple time points utilized the reciprocal principal component analysis (rPCA) method with ‘k.anchor=5’ to mitigate batch effects. Following normalization and scaling, the standard Seurat clustering pipeline was followed, involving these sequential steps: FindVariableFeatures, ScaleData, RunPCA, FindNeighbors (1 to 13 PCs), and FindClusters. SingleR was applied to eliminate non-macrophage/monocyte cells, such as dendritic cells, T cells, B cells, fibroblasts, and endothelial cells using the Immunological Genome Project (ImmGen) database (10, 11). Visualization employed uniform manifold approximation and projection (UMAP) via the RunUMAP function. Violin plots and UMAP plots, overlaying gene expression levels, were generated using Seurat. Differentially expressed genes (DEGs) for each cluster were identified through the FindAllMarkers function. CellChat (v2) (12) was used to identify cell-to-cell communication.

### 2.11 Pathways analysis

Gene set variation analysis (GSVA) and gene set enrichment analysis (GSEA) were conducted using GSVA (v1.46.0) and the fgsea (v1.18.0) library in R (13, 14). Canonical pathway enrichment was determined using hallmark pathways from the mouse Molecular Signatures Database (MSigDB). Enriched pathways were identified based on an adjusted p-value less than 0.05 and a positive normalized enrichment score (NES).

### 2.12 Statistics

Statistical tests were performed in GraphPad Prism v10. To identify differentially expressed genes (DEGs) within a subgroup in comparison to all other cells we used the nonparametric Wilcoxon rank-sum test. *P*<0.05 was considered statistically significant.

### 2.13 Data and code availability

The raw and processed scRNAseq data generated in this study has been deposited into the Gene Expression Omnibus (GEO) repository at the National Center for Biotechnology Information (NCBI). Access to the IM dataset is available via the accession number GSE254338, and the AM dataset is available via the accession number GSE262466. The scRNAseq analysis code are available from the authors upon reasonable request.

## 3 Results

### 3.1 *Schistosoma* exposure increases the number of CCR2^+^ IMs in the lungs

**Figure 1:**
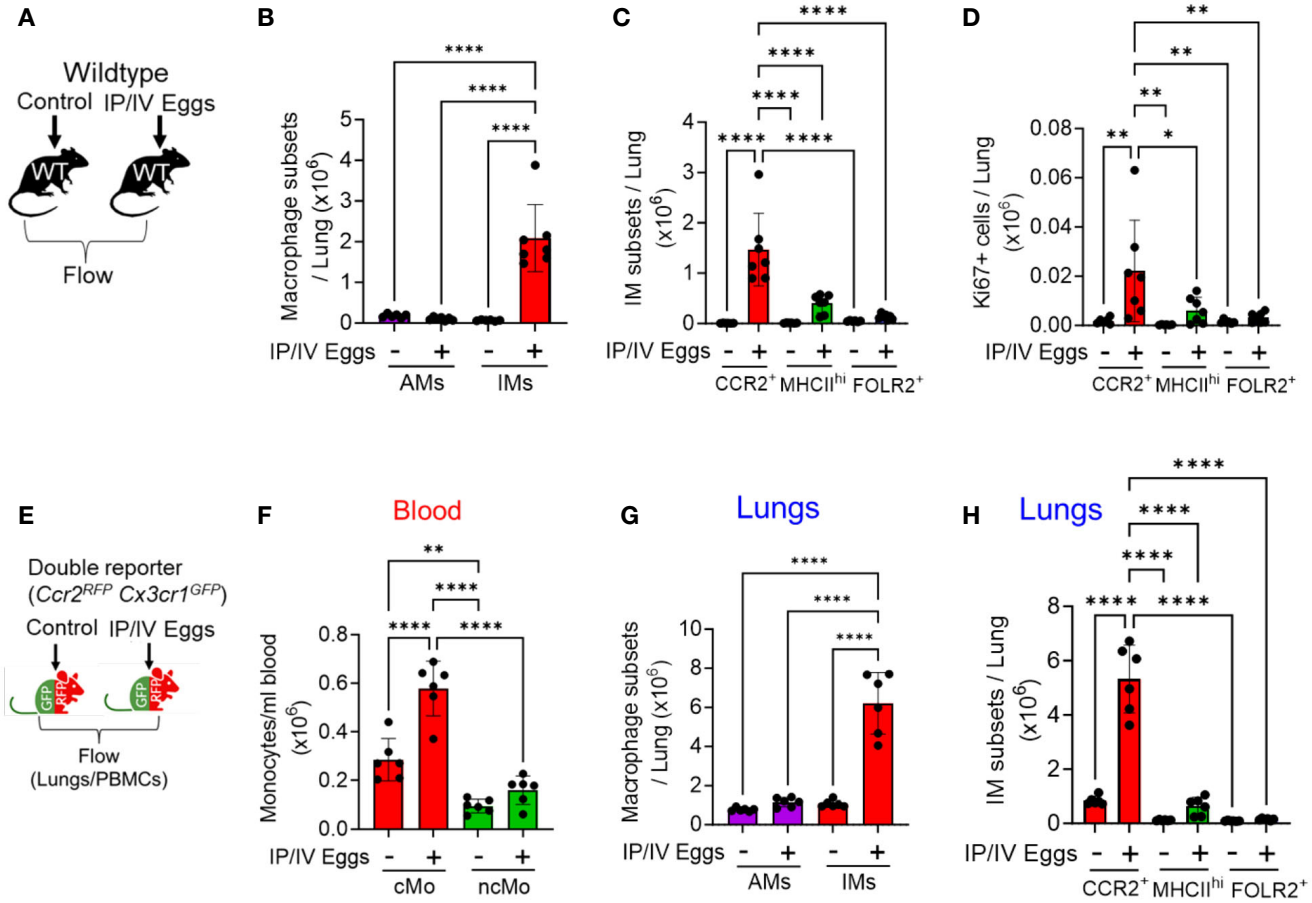
Increased number of CCR2^+^ IMs following *Schistosoma* exposure, 3 days after IV eggs. **(A)** Flow cytometry using wildtype mice was used to identify macrophage subpopulations wildtype mice: see gating strategy in **Supplementary Figures S1** and **S2**. Absolute number of **(B)** AMs and IMs number (n=6-7/group) and **(C)** IM subpopulations in wildtype mice (n=6-7/group). **(D)** Proliferation as identified by intracellular Ki67 expression among the IM subpopulations (n=6-7/group). **(E)** The findings were confirmed by flow cytometry using *Ccr2^RFP^Cx3cr1^GFP^* double reporter mice to identify intravascular and extravascular monocyte/macrophage subpopulations—see gating strategies in **Supplementary Figures S3 and S4** and **S4**, respectively. Quantitative analysis of the absolute number of **(F)** intravascular classical monocytes (cMo) and non-classical monocytes (ncMo; n=6/group). **(G)** Absolute number of pulmonary AMs and IMs (n=6/group). **(H)** Absolute number of the 3 IM subpopulations in the double reporter mice (n=6/group). **(B, G)** are 2-way ANOVA; for each, the variation from *Schistosoma* exposure is *P*<0.0001, the variation from AM versus IM is *P*<0.0001, and the interaction between the two factors is *P*<0.0001. The other statistical tests are 1-way ANOVA. Post-hoc Tukey test is shown throughout; *P* values: *P<0.05; **P<0.01; ****P<0.0001. IP, intraperitoneal; IV, intravenous; cMo, classical monocytes; ncMo, non-classical monocytes; AM, alveolar macrophages, IM, interstitial macrophages; WT, wildtype, GFP, green fluorescent protein; RFP, red fluorescent protein.

To identify pulmonary IM subpopulations in *Schistosoma*-exposed mice, we performed flow cytometry on single cell lung digests from wildtype mice, either control (unexposed) or *Schistosoma* sensitized and then intravenously (IV) challenged with *Schistosoma* eggs, 3 days after the IV eggs were administered (**Figure 1A**), using the gating strategy shown in **Supplementary Figure S1**. We observed a greater number of interstitial macrophages (IMs) but no significant change in alveolar macrophages (AMs) in the lungs of the mice following *Schistosoma* exposure (**Figure 1B**). To confirm the gating of IMs versus AMs, we added SiglecF, which is high in AMs and low in IMs, and validated that our IM gate was capturing only SiglecF^lo^ cells, whereas the AM gate was capturing predominantly Siglec^hi^ cells (**Supplementary Figures S2A-B**). This approach confirmed that IMs were increased but AMs were not following *Schistosoma* exposure (**Supplementary Figure S2C**).

Using flow cytometry, we identified 3 IM subpopulations, as have been previously described (15): (1) FOLR2^+^ resident IMs; (2) MHCII^hi^ resident IMs; and those that were both FOLR2^-^ and MHCII^lo^ were (3) CCR2^+^ recruited IMs. Of note, the previously description characterized one of the IM subpopulations as “TLF”, so-named as these cells express expressing TIMD4 and/or LYVE1 and/or FOLR2 (15). Among these 3 IM subpopulations, we found the CCR2^+^ subpopulation increased following *Schistosoma* exposure, with no change in the MHCII^hi^ or FOLR2^+^ subpopulations (**Figure 1C**). The additional gating strategy using SiglecF confirmed a significant increase in CCR2^+^ IMs, as well as an increase in MHCII^hi^ IMs (**Supplementary Figure S2D**). We assessed proliferation status by Ki67 expression, and observed greatest Ki67 expression in the CCR2^+^ IM subset following *Schistosoma* exposure (**Figure 1D**), suggesting this subpopulation was increasing in number through both recruitment and proliferation. We analyzed the Ly6c expression status of the CCR2^+^ IMs to gain insight if they may be derived from classical (cMo-Ly6c^hi^) or non-classical (ncMo-Ly6c^lo^) monocytes (as per gating in **Supplementary Figure S2**), and found the majority of the CCR2^+^ IMs were Ly6c^hi^, consistent with being derived from classical monocytes (**Supplementary Figure S2E**).

To confirm these observations and further characterize the IM subpopulations, we performed complementary flow cytometry using *Ccr2^+/RFP^Cc3cr1^+/GFP^* mice. These double reporter mice enable identifying CCR2-expressing cells by RFP expression, and CX3CR1 (fractalkine receptor)-expressing cells by GFP expression; CCR2 is a prototypical marker of classical monocytes, whereas CX3CR1 is a prototypical marker of non-classical monocytes (16), and the recruitment of each population is driven by ligands signaling through these receptors. Three days after intravenous *Schistosoma* egg challenge, we performed flow cytometry analysis on the peripheral blood and single cell lung digestions (**Figure 1E** and **Supplementary Figure S3**). We identified by quantitative analysis of the blood a significant increase in the number of circulating CCR2^hi^CX3CR1^lo^ cMos, with relatively little change in CCR2^lo^CX3CR1^hi^ ncMos (**Figure 1F**).

Flow cytometry of the lung using the *Ccr2^+/RFP^Cc3cr1^+/GFP^* reporter mice (using the gating in **Supplementary Figures S4A, B**) also confirmed an increase in IMs but not AMs following *Schistosoma* exposure (**Figure 1G**), and among the IM subpopulations only the CCR2^+^ IMs significantly increased following *Schistosoma* exposure (**Figure 1H** and **Supplementary Figures S5A-S5B**). Analysis of the CCR2^+^ IMs in control reporter mice revealed 2 subpopulations that appeared similar to the peripheral blood CCR2^hi^CX3CR1^lo^ cMos and CCR2^lo^CX3CR1^hi^ ncMos (**Supplementary Figure S3**), consistent with cells derived from these monocytes, and are now recruited IMs. In the *Schistosoma* exposed mice, the CCR2^+^ IMs were now largely CCR2^hi^CX3CR1^lo^, consistent again with recruited IMs being substantially derived from circulating cMos (**Supplementary Figure S5C**).

We also performed immunostaining of the lungs of *Ccr2^+/RFP^Cx3cr1^+/GFP^* reporter mice to identify cell location and phenotypes relative to airways and vessels. This demonstrated in control mouse lungs that CCR2-RFP cells are located in small pockets near lung vessels likely in the adventitial space, whereas CX3CR1-GFP cells are more broadly distributed as well as around airways (**Supplementary Figures S6A-C**). After *Schistosoma* sensitization and challenge, the numbers of both CCR2-RFP and CX3CR1-GFP cells substantially increase (**Supplementary Figures S6D-E**). Furthermore, some cells are observed to be expressing both CCR2-RFP and CX3CR1-GFP, which increase particularly in a perivascular distribution following *Schistosoma* exposure (**Figure S6F**). Comparing to our flow cytometry using *Ccr2^+/RFP^Cx3cr1^+/GFP^* reporter mice, we align Cx3cr1-GFP single positive cells largely with FOLR2+ IMs (**Supplementary Figure S7**). In contrast, both CCR2-RFP single positive and CCR2-RFP CX3CR1-GFP double positive cells appear to be a combination of both CCR2+ IMs and MHC2^hi^ IMs.

### 3.2 FOLR2^+^ IMs are a source of CCL2 in *Schistosoma*-PH

**Figure 2:**
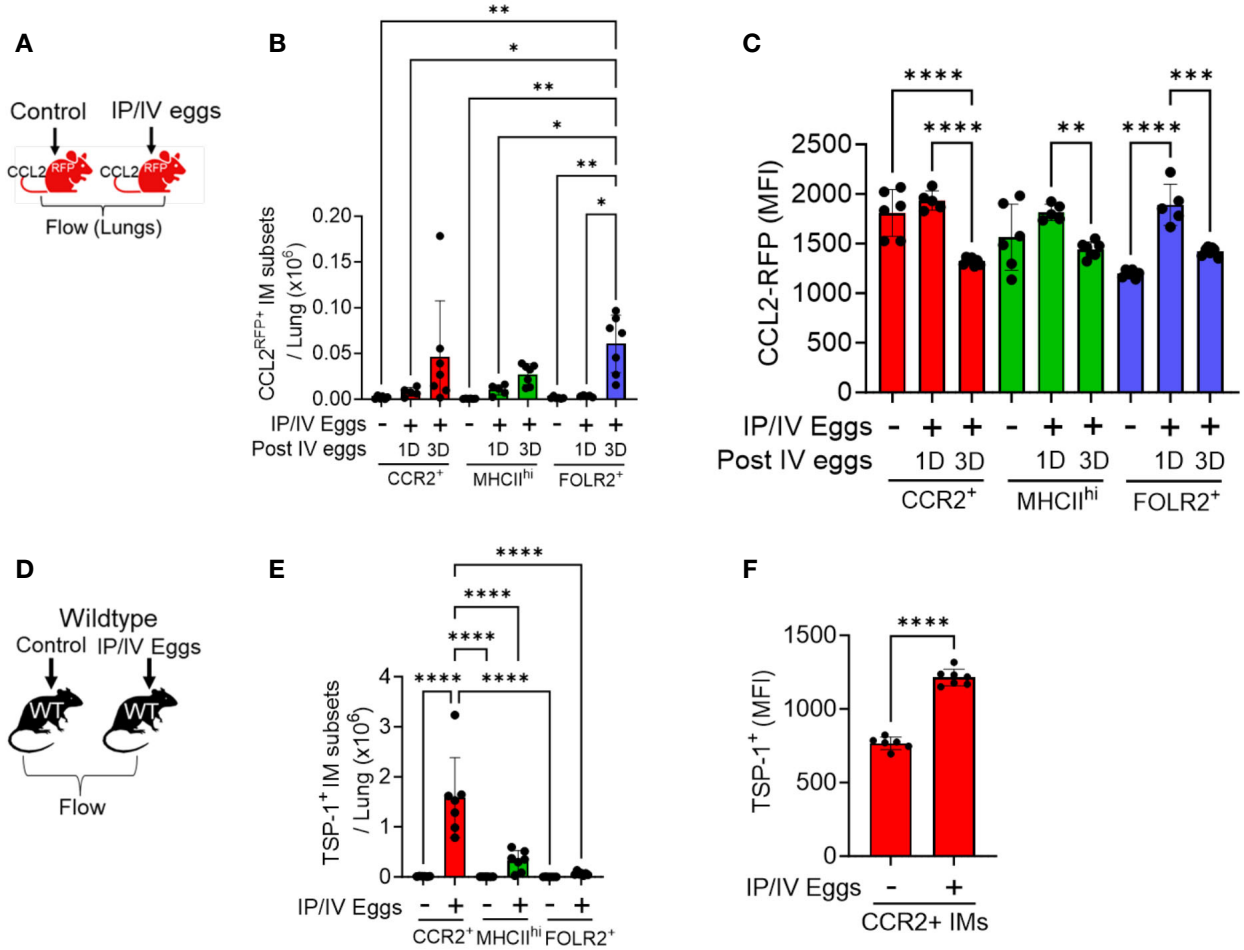
CCL2 and TSP-1 expression by IM subpopulations in *Schistosoma*-PH. Flow cytometry analysis using CCL2^RFP^ reporter mice following *Schistosoma* exposure to identify RFP expression in the 3 IM subpopulations: see gating strategy in **Supplementary Figure S4**. on **(A)** days 1 (N=3-5/group). and **(B)** 3 IM populations after IV *Schistosoma* egg challenge (N=6-7/group). **(C)** MFI of CCL2-RFP in the FOLR2^+^ IM subset on days 1 and 3 after IV *Schistosoma* egg challenge. **(D)** Absolute number of cells intracellular TSP-1^+^ in wildtype mice by flow cytometry of the 3 IM subpopulations, 3 days after IV eggs (N=7/group). **(E)** MFI of intracellular TSP-1 expression in CCR2^+^ IMs (N=6-7/group). IP, intraperitoneal; IV, intravenous; IM, interstitial macrophages; RFP, red fluorescent protein; MFI: mean fluorescence intensity. T-test or ANOVA with post-hoc Tukey test; P-value: *P<0.05; **P<0.01; ***P<0.001; ****P<0.0001.

The recruitment of classical monocytes requires a gradient of recruitment ligands emanating from inflamed tissue, and these cytokines CCL2, 7 and 12 are all present at increased concentrations in whole-lung lysates of *Schistosoma*-PH mice (3). To investigate the cellular source, we performed flow cytometry at 1 and 3 days after *Schistosoma* egg exposure using CCL2^RFP^ reporter mice, in which CCL2-expressing cells also express RFP, using the gating strategy shown in **Supplementary Figure S8**. This approach identified an increase in CCL2-expressing cells among all 3 IM subpopulations, with the greatest increase in FOLR2^+^ IMs (**Figures 2A, B**), most notably 3 days after IV egg administration. The FOLR2^+^ IMs (the dominant IM subpopulation at baseline—**Supplementary Figure S5B**) had relatively low baseline CCL2 expression as quantified by median fluorescent intensity (MFI), which then increased significantly as early as 1 day after *Schistosoma* exposure (**Figure 2C**). In contrast, the CCL2 expression in the other populations was higher at baseline but did not further increase following *Schistosoma* exposure at day 1. Between days 1 and 3, the CCL2 expression deceased in all IM subpopulations. These data indicate that the FOLR2+ IM subpopulation is a major source of CCL2 through a combination of increased cell numbers and increased expression by each cell. We also studied CCL2 expression in stromal cells, and observed *Schistosoma* exposure increased CCL2 expression by fibroblasts, another resident cell population (**Supplementary Figure S9**).

### 3.3 Recruited IMs are a source of TSP-1 in *Schistosoma* PH

In order to identify the major cellular sources of TSP-1, we performed flow cytometry on wildtype mice with intracellular staining for TSP-1, using the gating strategy shown in in **Supplementary Figure S10**. Quantitative analysis of the IM subpopulations revealed that CCR2^+^ IMs are a major source as they were present in larger absolute numbers and exhibited significantly higher MFI for TSP-1 after *Schistosoma* exposure (**Figures 2D-F**). We also observed increased TSP-1 expression by AMs, but no difference in TSP-1 expression by endothelial cells or fibroblasts (**Supplementary Figure S11**).

### 3.4 scRNAseq identifies 3 IM subpopulations which retain their identity after *Schistosoma* exposure

To further investigate the phenotype of IMs in *Schistosoma*-induced inflammation and PH, lung macrophages were flow-sorted and characterized by scRNAseq using *Cx3cr1^gfp/+^* mice, from either unexposed controls or at 1, 3 and 7 days after IV *Schistosoma* egg exposure, with 1 male and 1 female mouse in each of these 4 groups (**Supplementary Figure S12**). Using a Cx3cr1 reporter mouse enabled identifying inflammatory macrophages which are potentially biased to an M2 phenotype (17, 18) (as would be expected with *Schistosoma*-exposure) with a significantly bright signal as is required for accurate sorting of macrophages which are prone to autofluorescence (19). Of note, the scRNAseq experiments were performed at Denver elevation, whereas the preceding flow cytometry experiments were performed at sea level.

**Figure 3:**
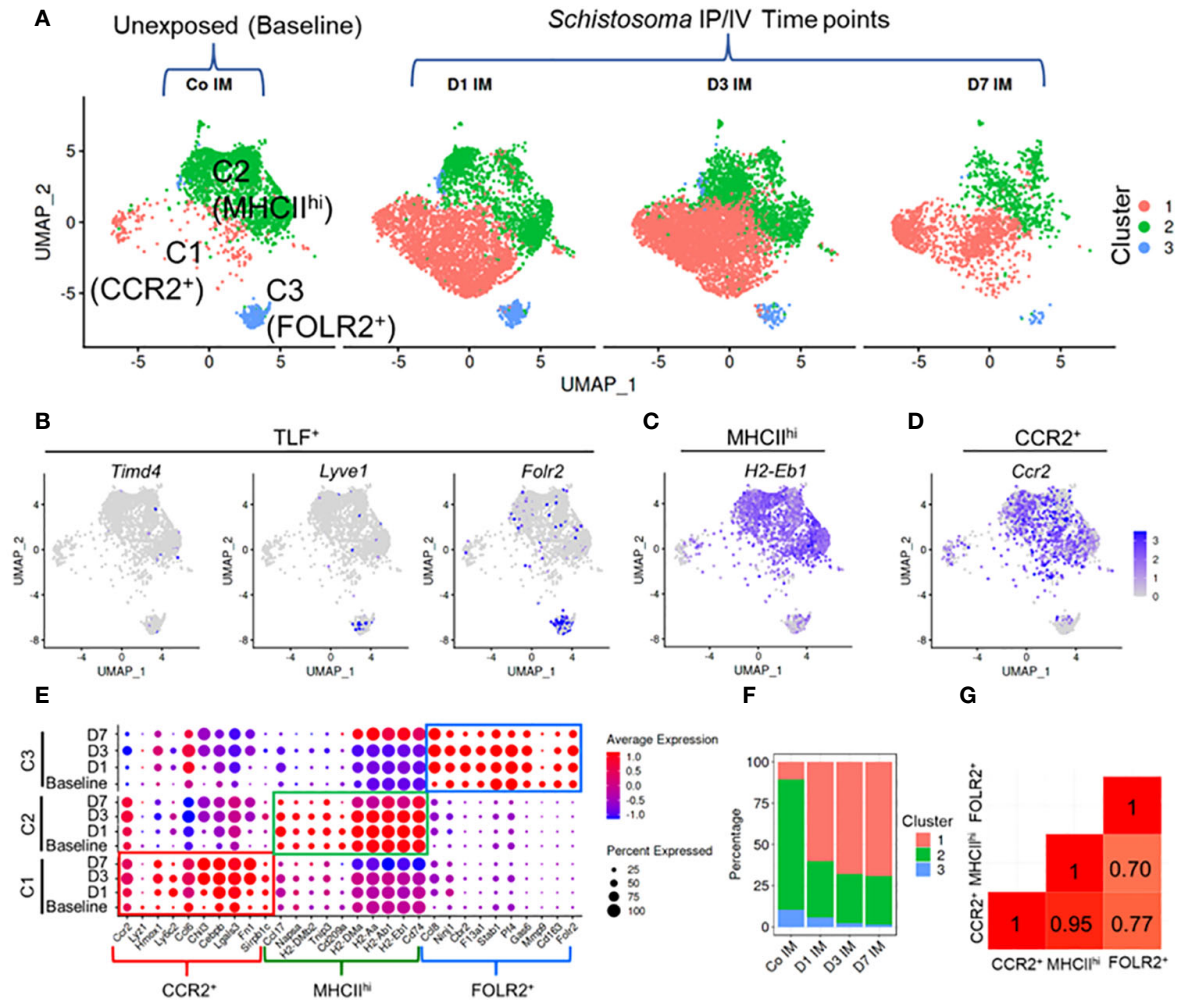
Single cell RNAseq characterization of IMs in *Schistosoma* induced inflammation. **(A)** UMAP plot of 3 IM clusters in control mice, and at 1, 3 and 7 days after *Schistosoma* exposure. **(B)** Expression profiles of the Timd4, Lyve1, and Folr2 genes, which mark the TLF^+^ cluster. Expression profiles of **(C)** H2-Eb1 (which spans both the MHCII^hi^ and CCR2^+^ subpopulations) and **(D)** CCR2 (which is more uniquely expressed by the CCR2^+^ subpopulation). **(E)** Expression of key gene signatures at baseline at 1, 3 and 7 days after *Schistosoma* exposure in the 3 identified IM subpopulations, demarcated CCR2^+^, MHCII^hi^, and FOLR2^+^. **(F)** Relative percentage of the 3 clusters at each timepoint. **(G)** Correlation analysis using the Pearson method between the 3 subpopulations. IP, intraperitoneal; IV, intravenous; IM, interstitial macrophages; **(D)** with numerical value indicates number of days after *Schistosoma* IV challenge.

A total of 17,926 cells were analyzed across all 4 groups. Following quality control, including removal of doublets and damaged cells, 14,665 cells were identified, and subjected to clustering analysis. Using Immunological Genome Project (ImmGen) data to identify different immune cell populations, we retained macrophages and monocytes which were relatively similar in the UMAP plot, and excluded stromal and other immune cells, such as endothelial cells and dendritic cells (**Supplementary Figure S13**). In the unexposed mice, under homeostatic conditions, this approach revealed three distinct IM clusters (**Figure 3A**), which could be identified in a manner similar to prior publications (15) as CCR2^+^, MHCII^hi^, and TLF^+^ (or FOLR2^+^–the terminology we use here, for comparison to the flow cytometry data above; **Figures 3B-D**). Notably, the top genes expressed by these populations had high similarity with the top genes expressed by the 3 IM subpopulations previously identified by *Dick et al.* (15) (**Supplementary Figure S14**).

Following cluster identification, we examined changes in the three IM clusters following *Schistosoma* exposure. Notably, each of the 3 IM subpopulations closely retained their core distinguishing gene expression patterns at 1, 3 and 7 days following *Schistosoma* exposure (**Figure 3E**). Quantitatively, there was a substantial increase in CCR2^+^ IMs starting at day 1 post-exposure, which persisted (**Figure 3F**). The relative increase in CCR2^+^ IMs was accompanied by a relative decrease in the MHCII^hi^ and FOLR2^+^ IM subpopulations. However, this is likely a dilution artifact from the influx of CCR2^+^ IMs (as in **Supplementary Figure S5B**) rather than a true reduction in the number of these cells, as by flow cytometry we found an 178.8-fold increase in CCR2+, 39.1-fold increase in MHCIIhi, and 2.8-fold increase in FOLR2+ IMs following *Schistosoma* exposure (**Figure 1C**).

Comparison of gene expression between subpopulations identified a significant transcriptional difference in the FOLR2^+^ subpopulation from MHCII^hi^ and from CCR2^+^ IMs, whereas there was a relatively high degree of correlation suggesting shared gene profiles between MHCII^hi^ and CCR2^+^ IMs (**Figure 3G**). We distinguished cells derived from male versus female mice by identifying cells expressing Y-chromosome genes and X-linked inactivation genes, and found no difference in IM distribution between sexes (**Supplementary Figure S15**).

### 3.5 FOLR2^+^ IMs express CCLs 2, 7 and 12, whereas CCR2^+^ IMs express TSP-1

**Figure 4:**
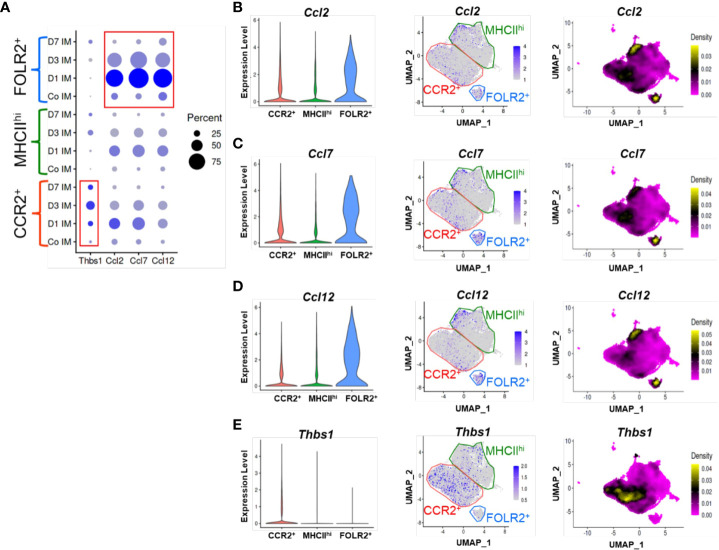
TSP-1 and CCR2 ligand expression in IM subpopulations by scRNAseq. **(A)** Cluster based expression of the CCR2 ligands Ccl2, Ccl5, and Ccl12 and Thbs1 (the gene encoding TSP-1) in IMs from controls and at days 1, 3 and 7 following *Schistosoma* exposure. The red boxes identify expression of *Thbs1* in the CCR2^+^ IM subpopulation, and CCR2 ligands in the FOLR2^+^ IM subpopulation. **(B-E)** Violin plots, features plots and density plots showing the expression of *Ccl2*, *Ccl7*, *Ccl12* and *Thbs1* by the IM clusters.

Using the scRNAseq dataset, we examined the expression of the CCR2 ligands CCLs 2, 7 and 12 across the three IM clusters. At baseline there was relatively little expression of these ligands by any of the clusters (**Figure 4A**). Following *Schistosoma* challenge there was a significant increase in expression of all 3 transcripts particularly in the FOLR2^+^ subpopulation (**Figures 4B–D**), with greatest expression on day 1 after IV eggs, which then decreased but remained above baseline (**Figure 4A**). There was also a small subpopulation of MHCII^hi^ IMs which expressed CCLs 2, 7 and 12 (**Figures 4B–D**).

Analogously, we examined Thbs1 expression (the gene encoding TSP-1), and found little baseline expression among any clusters (**Figure 4A**). After *Schistosoma* challenge, Thbs1 expression significantly increased among all clusters, and particularly in the CCR2^+^ subpopulation (**Figure 4E**), again closely mirroring the flow cytometry results. Among CCR2^+^ IMs, TSP-1 expression was maximal at day 3, but was also higher than baseline at days 1 and 7 (**Figure 4A**). As CCR2^+^ IMs are the dominant subpopulation following *Schistosoma* exposure (**Figure 3C**), the majority of TSP-1 expression is likely from CCR2^+^ IMs.

### 3.6 IM subpopulation have distinct molecular identities by inflammatory markers and putative cell-to-cell signaling

**Figure 5:**
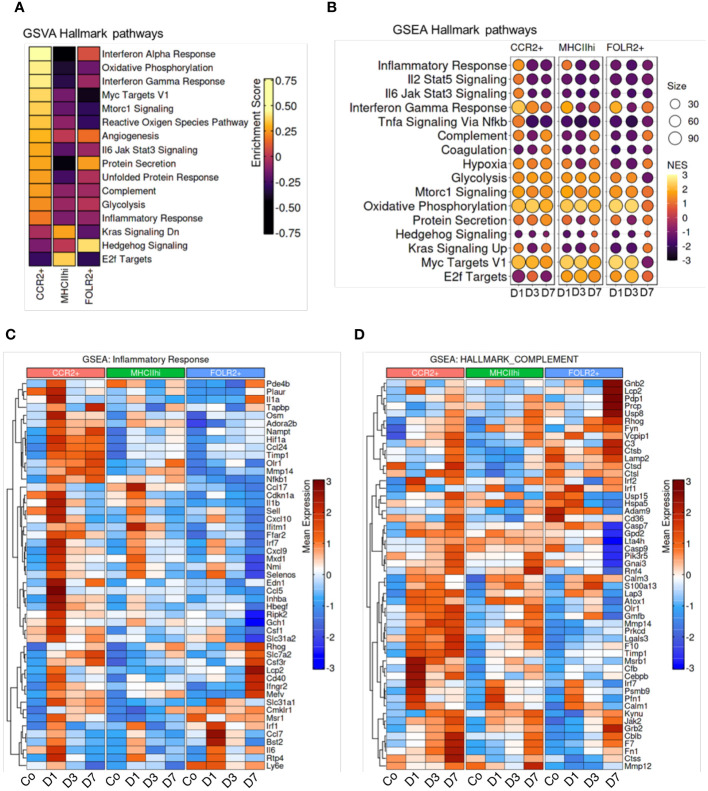
Enrichment of hallmark pathways in response to *Schistosoma* exposure. **(A)** Gene Set Variation Analysis (GSVA) between subpopulations, and **(B)** Gene Set Enrichment Analysis (GSEA) across timepoints demonstrates cluster and temporal based hallmark pathway activation. Genes associated with the CCR2+, MHCII^hi^, and FOLR2+ IMs show differences in the gene sets of **(C)** inflammatory response and **(D)** complement in different subpopulations at different timepoints.

To investigate molecular functions of the CCR2^+^, MHCII^hi^, and FOLR2^+^ immune cell subpopulations, we employed unbiased gene set variation analysis (GSVA) and gene set enrichment analysis (GSEA) focusing on hallmark pathways. GSEA identifies differential expression in predefined gene sets between groups, while GSVA assesses variation in gene set activity within individual samples. GSVA revealed specific functional pathway enrichments in distinct subpopulations. In the CCR2^+^ subpopulation, there was enrichment in pathways related to the inflammatory response, IL6-Jak-Stat3 signaling, interferon gamma response, complement, oxidative phosphorylation and glycolysis (**Figure 5A**). The MHCII^hi^ cluster showed enrichment in E2f targets. The E2F-targets encompass numerous genes responsible for initiating and advancing DNA replication, DNA repair, and regulating chromatin. Cell cycle advancement is pivotal for cell proliferation, and the proliferation of smooth and endothelial cells contributing to vascular remodeling is a hallmark of *Schistosoma*-PH. The FOLR2^+^ subpopulation exhibited enrichment in hedgehog and protein secretion pathways. Consistent with the GSVA results, GSEA demonstrated higher enrichment in inflammatory pathways at day 1 following *Schistosoma* exposure (**Figure 5B**). These findings suggest that the initiation of an inflammatory response might be a prerequisite for subsequent remodeling events. This is supported by our observation that blocking Th2 inflammation did not induce the PH phenotype. Other pathways varied across clusters, such as oxidative phosphorylation and glycolysis, reaching maximum enrichment at days 1 and 3 (**Figure 5B**). Of note, elevated oxidative phosphorylation and glycolysis in the lungs may promote monocyte recruitment and proliferation, potentially fueling inflammatory responses and tissue remodeling. This correlation underscores the interplay between metabolic activity and immune processes in lung physiology. Gene expression profiles for specific genes within the inflammatory response and complement pathways are shown in **Figures 5C, D**, respectively. The gene-specific effects in the PI3K-AKT-mTOR signaling, protein secretion, glycolysis, and hypoxia hallmark pathways among CCR2+, MHCII^hi^, and FOLR2+ IMs across the different timepoints are shown in **Supplementary Figure S15**. Interestingly, the CCR2+ subpopulation exhibited remarkably higher expression of inflammation particularly on day 1 and complement on all days (**Figures 5C, D**), and PI3K-AKT-mTOR signaling, protein secretion, glycolysis and hypoxia-associated signatures on all days following *Schistosoma* exposure (**Supplementary Figure S16**). These signatures in the CCR2+ subpopulation following Schistosoma exposure suggests potential involvement of these pathways in mediating monocyte recruitment and inflammatory responses in the lung. In contrast, the FOLR2^+^ subpopulation displayed distinct gene signatures related to inflammation and complement (**Figures 5C, D**), protein secretion after *Schistosoma* exposure (**Supplementary Figure S16B**). These observed in the FOLR2+ subpopulation following *Schistosoma* exposure hint at potential mechanisms underlying its specialized role in immune response modulation and tissue homeostasis regulation.

We identified putative cell-to-cell communication using complementary ligand and receptor expression. This suggested the greatest communication was occurring between FOLR2^+^ IMs and CCR2^+^ IMs, with the FOLR2^+^ IMs the sender of communication expressing multiple ligands of CC chemokines pathways (such as CCL2, CCL6, CCL6 and CCL12) and the CCR2^+^ IMs the recipients of communication (expressing receptors; **Supplementary Figures S17A and B**). The greatest ligand expression occurred on day 1 after *Schistosoma* exposure (**Supplementary Figure S17C**).

We performed directed analysis for inflammatory patterns in the IM subpopulations, and found unique features of Type 2 inflammation within each of the 3 IM subpopulations (**Supplementary Figure S18**), with distinct Type 2 ligands expressed by each subpopulation. On day 1 after *Schistosoma* challenge, the CCR2^+^ population expressed Arg1, Il5 and Il13; whereas the MHCII^hi^ population expressed Il4, Il5, Il13 and Ccl17; and the FOLR2+ expressed Il10 and Postn. The cytokine receptors were also distinct: the MHCII^hi^ population expressed Il13ra1, whereas the FOLR2^+^ population expressed IL4ra, and the CCR2^+^ population expressed neither to any significant degree.

We compared the key markers characterizing IM clusters in hypoxia-induced PH following exposure to a simulated altitude of 5,486 meters, a very different stimulus: hypoxia prototypically drives a Type 1 immune response (20), whereas *Schistosoma* drives a Type 2 immune response. Despite these differences, both hypoxia and *Schistosoma* cause experimental and human PH. Work in the hypoxia PH system identified 4 IM subpopulations, characterized as MHCII^hi^CCR2^+^EAR2^+^, TLF^+^VCAM1^hi^, MHCII^hi^CCR2^+^EAR2^-^, and TLF^+^VCAM1^lo^ (21). Comparing these 4 IM subpopulations in hypoxic-PH to the 3 populations in *Schistosoma*-PH identified that the *Schistosoma*-PH CCR2^+^ IMs were analogous to the hypoxia-PH MHCII^hi^CCR2^+^EAR^+^ IMs; the *Schistosoma*-PH MHCII^hi^ IMs were analogous to the hypoxia-PH MHCII^hi^CCR2^+^EAR^-^ IMs; and the *Schistosoma*-PH FOLR2^+^ IMs were analogous to a combination of the TLF^+^VCAM^hi^ and TLF^+^VCAM^lo^ IMs in the hypoxia-PH model (**Supplementary Figure S19**).

### 3.7 Alveolar macrophages also demonstrate significant inflammatory phenotypes

AMs were also flow sorted and subject to scRNAseq analysis. This approach identified 4 AM subpopulations which were present at all timepoints (**Supplementary Figure S20A**). Of these, AM1 was characterized by LysM and CD9 expression; AM2 was characterized by Mmp12 and Hmox1 expression; AM3 was characterized by Retnla expression; and AM4 was characterized by expression of complement factors and MHCII (**Supplementary Figure S20B**). In control mice, the dominant AM subpopulation was AM1 (**Supplementary Figure S20C**). Following Schistosoma exposure, the AM2 subpopulation relatively increased peaking at day 3, and the AM4 subpopulation was maximal at day 7 (**Supplementary Figure S20C**). Comparing male versus female mice across all timepoints, female mice and relatively more AM2 and male mice had relatively more AM1 cells (**Supplementary Figure S20D**).

We analyzed at the expression of the CCR2 receptor cytokines CCL2, 7 and 12 in the AM subpopulations. There was modest expression of Ccl12 by the AM4 subpopulation on day 1, whereas Ccl7 was expressed by the AM1, AM2 and AM3 subpopulations particularly on day 7 (**Supplementary Figure S21**). The gene encoding TSP-1, Thbs1, was also expressed by the AM1, AM2 and AM3 subpopulations on day 7. However, the relative expression of all of these mRNAs by AMs was substantially lower than the expression by IM subpopulations (**Supplementary Figure S21A**).

Pathway analysis was conducted on the 4 AM subpopulations to identify hallmark pathways of each subpopulation. This found similarities and differences between the subpopulations: TNF-alpha signaling via NF-kB was present in AM1 and AM3; complement signaling and oxidative phosphorylation was present in AM1, AM2 and AM4; and IFN-gamma response was present in AM2 and AM4 (**Supplementary Figure S22**).

## 4 Discussion

Schistosoma exposure induces a robust Type 2 immune response, which is capable of causing experimental and human PH. Here, using a combination of flow cytometry and scRNAseq, coupled with reporter mice to more robustly characterize cell populations, we observed a complex interplay between distinct IM subsets in the pathogenesis of Schistosoma-induced PH. We observed one set of immunity-stimulated IMs produces monocyte recruitment ligands, and a separate subset of IMs (derived from recruited monocytes) expresses TSP-1. We previously observed that IMs contribute to the pathogenesis of Schistosoma-induced PH via expression of TSP-1: functionally, TSP-1 activates TGF-β which results in vascular remodeling (3). These TSP-1-expressing IMs are derived from circulating monocytes, as blocking monocyte recruitment by CCR2 deletion in the bone marrow compartment blocks recruitment of these cells, and protects mice from experimental Schistosoma-PH (3).

Recent publications have characterized 2 resident IM subpopulations by scRNAseq and surface markers, which are shared across tissue compartments (15, 22). These cells are considered resident because they remain in the tissue, and have been termed MHCIIhi and TLF+ (15). Dick et al. (15) used the “TLF” abbreviation as these cells express TIMD4 and/or LYVE1 and/or FOLR2; in the Schistosoma model, we found FOLR2 functioned well as the primary marker of this population. There has also been characterized a recruited IM subpopulation, identified by CCR2 expression, that transiently enters and departs from the circulation, and is derived from circulating monocytes (15, 22–24). We identified the same populations in our model, and interrogated how they change in the inflammatory state induced by *Schistosoma* exposure. Remarkably, we observed the same 3 populations to be present in *Schistosoma*-challenged mice, albeit in different proportions, and with new mRNA and proteins being expressed by the cells consistent with activated phenotypes. By flow cytometry we found marks of both nonclassical and classical monocytes contributing to the CCR2+ IM subpopulation, but classical monocytes dominated after *Schistosoma* exposure.

Pathologic mechanisms of monocyte recruitment and IMs are also present in hypoxia-induced PH (3, 7, 25–29). There is a high degree of similarity in the IM populations observed in both hypoxic- and Schistosoma-PH (21), although hypoxia induces a Type 1 inflammatory phenotype in IMs whereas *Schistosoma* induces a Type 2 inflammatory phenotype. scRNAseq analysis of the hypoxia-PH model revealed 2 IM subpopulations directly analogous to the CCR2+ and MHCII^hi^ IM subpopulations in Schistosoma-PH (21). Prolonged hypoxia up to 21 days drove the development of 2 TLF IM subpopulations (21), which are similar to the single *Schistosoma*-PH FOLR2+ population only studied up to 7 days.

Macrophages in the setting of Type 2 immunity have been various termed “alternatively activated” or “M2” (30, 31), and further subdivided into M2a, M2b, etc. (32), although this nomenclature has been criticized as largely based on *in vitro* models with artificial stimuli and the exact phenotypes are likely to be more intersectional and fluid (33, 34). Here, utilizing a robust Type 2 antigen, we found each of the 3 IM subpopulations exhibited a unique phenotype including aspects of prototypical Type 2 immunity. Only CCR2^+^ IMs expressed Arginase1; only FOLR2^+^ IMs expressed IL-10; only MHCII^hi^ IMs expressed IL-4; and both CCR2^+^ and MHCII^hi^ but not FOLR2^+^ IMs expressed IL-5 and IL-13. Prior work has also identified functionally heterogenous macrophage populations in schistosomiasis (35), together suggesting a complex network of interactions. We have previously observed that IL-4/IL-13 expression by CD4 T cells is required for Schistosoma-induced PH (36); other cells such as macrophages and eosinophils (37) can also express IL-4, and we suspect non-T cell function as downstream amplifiers of the Type 2 immune response.

Understanding the precise phenotypes and roles of discrete IM subpopulations is vital to dissect the disease process and identify novel therapeutic approaches in inflammatory vascular diseases (29, 38). We have previously targeted discrete pathologic cytokines, including IL-4, IL-6, IL-13, and TSP-1, finding that they all contribute to *Schistosoma*-induced PH (2–4, 6, 39). Here we focus on signaling pathways via cytokines between cell populations which underlie *Schistosoma*-PH pathogenesis. Specifically, a mechanism is required which transduces the IL-4 and IL-13 expressed by CD4 T cells to cause recruitment of TSP-1^+^ IMs, resulting in the pulmonary vascular pathology. We previously observed that CCR2^-/-^ mice are relatively protected from *Schistosoma*-induced PH, including suppression of TSP-1^+^ IM recruitment (3). Recruitment of circulating monocytes to become TSP-1^+^ IMs would thus be mediated by tissue expression of CCR2 ligands, namely cytokines CCL2, 7 and 12. This premise suggests that there is at least 1 cell population that: a) expresses IL-4/13 receptors, b) has evidence of Type 2 immune activation, and c) expresses CCLs 2, 7 and/or 12 in the context of *Schistosoma* exposure. Here, we identify that FOLR2+ IMs have these characteristics and are likely to functionally serve in this role, as they: a) express the common IL-4/13 receptor IL4Ra; b) express periostin, an IL-4/13 target (40) which we previously found upregulated in *Schistosoma*-PH mice and humans (2); and c) express CCLs 2, 7 and 12.

We also observed in the scRNAseq dataset a number of pathways that were enriched following Schistosoma exposure, including IL6-Jak-Stat3 signaling, PI3K-AKT-mTOR signaling, interferon gamma response, complement, oxidative phosphorylation, glycolysis, and hypoxia. Some of these findings corroborate prior observations, as we previously found a role for HIF1a and HIF2a signaling in macrophages in promoting *Schistosoma*-induced PH (3), whereas IL6 and STAT3 appear to be protective in *Schistosoma*-PH (39). Complement is known to be activated in schistosomiasis (41), and we currently have an ongoing project studying the mechanistic role of complement in *Schistosoma*-PH. Other pathways uncovered here will be investigated in future studies to determine their functional nature in this disease model.

A limitation of our work is that we have not functionally determined the necessity of CCL2/7/12 expression by FOLR2+ IMs. There could be other cell populations functioning as transducers in parallel, such as fibroblasts. We have previously observed an increase in perivascular fibroblast density in mice following repeated exposure to *Schistosoma*, associated with an increase in perivascular fibrosis and a persistent PH phenotype (5). AMs phenotypes may also cause pulmonary vascular disease, although the adjacency of IMs to the pulmonary vasculature suggests IMs are more likely to contribute the vascular disease. Bronchoalveolar lavage (BAL) can be used to sample AMs and other airway cells including those in the proximate airways, and can be used as a less invasive approach than lung tissue collection (42, 43); here, however, we employed flow cytometry on digested lung tissue already collected for IM analysis to characterize AMs as well, distinguishing the 2 populations based on cell surface markers. There could also be combinations of cells functioning together, such as one that receives IL-4/IL-13 signaling and communicates with a second cell–and this second cell releases CCLs 2/7/12. It could also be true in a robustly inflammatory condition such as schistosomiasis that suppressing any single pathway or cell may be inadequate to block the inflammatory cascade, as there are alternative pathways that compensate. In contrast, we previously observed that precisely targeting TSP-1 and TGF-β signaling, which is downstream of the Type 2 inflammation is sufficient to suppress experimental *Schistosoma*-PH.

Of note, the scRNAseq studies were done at Denver elevation, whereas the flow cytometry studies were done at sea level elevation. The SchPH phenotype appears to be similar at both elevations (3, 5). There are likely to be some differences due to mild hypoxia in cell physiology including IM phenotype with the 1500m elevation change, which is equivalent to a decrease in FiO2 from 21% to 19%. A comparison of scRNAseq data performed at sea level and Denver elevation reveals transcriptomic differences in oxidative phosphorylation and reactive oxygen species pathways (21).

In summary, we observed that following *Schistosoma* exposure, a subset of Type 2 immunity-stimulated IMs produces monocyte recruitment ligands, and a separate subset of IMs expresses TSP-1, thereby working together in concert to each distinctly contribute to PH pathogenesis.

## Data availability statement

The datasets presented in this study can be found in online repositories. The names of the repository/repositories and accession number(s) can be found below: GSE254338 and GSE262466 (GEO).

## Ethics statement

The animal study was approved by University of Colorado Anschutz Medical Campus IACUC and University of California San Francisco IACUC. The study was conducted in accordance with the local legislation and institutional requirements.

## Author contributions

RK: Conceptualization, Methodology, Writing – original draft, Writing – review & editing, Data curation, Investigation, Software, Visualization. SK: Data curation, Methodology, Writing – review & editing, Formal analysis. CM: Data curation, Investigation, Writing – review & editing, Formal analysis, Methodology. DF: Data curation, Investigation, Writing – review & editing. KN: Data curation, Investigation, Writing – review & editing. ML: Data curation, Investigation, Writing – review & editing. LS: Data curation, Investigation, Writing – review & editing. JN: Writing – review & editing, Formal analysis, Methodology. AM: Methodology, Writing – review & editing, Formal analysis. RT: Formal analysis, Writing – review & editing, Data curation, Investigation. KS: Data curation, Investigation, Methodology, Project administration, Writing – review & editing. BG: Conceptualization, Formal analysis, Funding acquisition, Methodology, Project administration, Resources, Supervision, Writing – original draft, Writing – review & editing.
