# Supplementary material for: Interstitial macrophage phenotypes in Schistosoma-induced pulmonary hypertension
Source: Front Immunol. 2024 May 8;15:1372957. doi: 10.3389/fimmu.2024.1372957 (PMC11109442; doi:10.3389/fimmu.2024.1372957)
Supplement: Supplementary file 1 [file DataSheet_1.docx]

Supplementary Material

## Supplementary Tables

| **Antibody Specificity** | **Fluorochrome** | **Clone** | **Final Concentration** | **Manufacturer** |
| --- | --- | --- | --- | --- |
| anti-mouse CD16/32 (blocking Fc domain) | - | 93 | 1μg | BioLegend |
| anti-mouse CD3 | eFluo450 | 17A2 | 0.5μg | eBioscience® |
| anti-mouse CD19 | eFluo450 | 1D3 | 0.5μg | eBioscience® |
| anti-mouse NK1.1 | eFluo450 | PK136 | 0.5μg | eBioscience® |
| anti-mouse Ly6G | eFluo450 | 1A8 | 0.5μg | eBioscience® |
| Fixable Viability dye | eFluo450 |  | 0.0625μg | eBioscience® |
| anti-mouse CD45 | AF700 | 30 F11 | 1μg | eBioscience® |
|  | BV570 | 30 F11 | 1μg | BioLegend |
|  | BV785 | 30 F11 | 0.3μg | BioLegend |
|  | APC-R700 | 30 F11 | 0.5μg | BD Biosciences |
| anti-mouse CD11b | SB600 | M1/70 | 0.3μg | eBioscience® |
|  | FITC | M1/70 | 0.125μg | BioLegend |
| anti-mouse CD64 | PerCP/eFluor 710 | X54-5/7.1 | 1μg | eBioscience® |
|  | APC | X54-5/7.1 | 0.8μg | BioLegend |
|  | PE/Cyanine7 | X54-5/7.1 | 0.5μg | BioLegend |
| anti-mouse CD11c | PE/Cyanine7 | N418 | 0.5μg | BioLegend |
|  | APC | N418 | 0.3μg | BioLegend |
| anti-mouse Ly6C | BV650 | HK1.4 | 0.2μg | BioLegend |
| anti-mouse I-A/I-E | SB700 | M5/114.15.2 | 0.15μg | eBioscience® |
| anti-mouse FOLR2 | APC | 10/FR2 | 0.6μg | BioLegend |
| anti-mouse CCR2 | BV510 | SA203G11 | 1μg | BioLegend |
| anti-mouse Ki-67 | PE/Cyanine7 | SolA15 | 0.4μg | eBioscience® |
|  | AF700 | 16A8 | 0.5μg | BioLegend |
| anti-mouse TSP-1 | PE | TX17.10 | 0.4μg | Novus |
| anti-mouse CCL2 | FITC | 2H5 | 0.3μg | eBioscience® |
| anti-mouse CD31 | APC | 390 | 0.5μg | BioLegend |
| anti-mouse Podoplanin (gp-38) | PE/Cyanine7 | 8.1.1 | 0.5μg | BioLegend |
| anti-mouse Siglec-F | PE-CF594 | E50-2440 | 0.5μg | BD Biosciences |

**Supplementary** **Table 1: Antibodies used in the mouse flow cytometry experiments.**

## Supplementary Figures


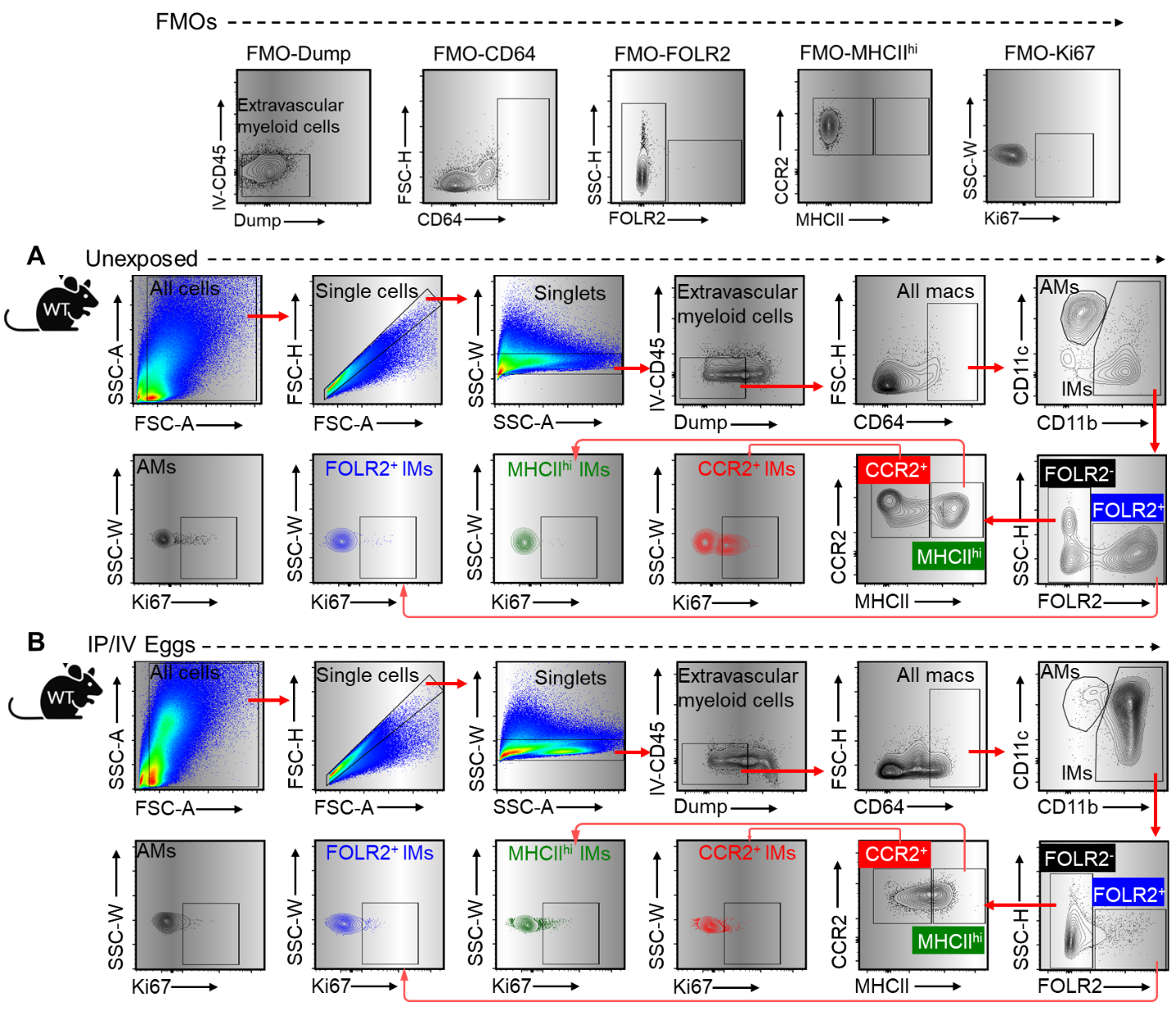


**Figure** **S1: Schematic representation of flow cytometry gating of pulmonary IM subpopulations in unexposed and *Schistosoma*-exposed wildtype mice**. Gating strategy to characterize IM subpopulations in (**A**) control and (**B**) *Schistosoma*-exposed wildtype mice. First singlets were gated negative for intravascular anti-CD45 and Lin-, and then gated CD64+ to identify macrophages. The macrophages were differentiated into AMs and IMs based on CD11b and CD11c expression. The IMs were subdivided by FOLR2 expression into FOLR2^+^ IMs and FOLR2^-^ cells. The FOLR2^-^ population was then subdivided by CCR2 and MHCII expression as CCR2^+^ or MHCII^hi^ IMs. Nuclear Ki67 expression was used to identify actively proliferating cells. FMOs are presented in the top rows. IP, intraperitoneal; IV, intravenous; IM: interstitial macrophage; AM: alveolar macrophage; FOLR2, folate receptor 2, SSC-A, side scatter area; FSC-A, forward scatter area; FSC-W, forward scatter width. These are representative images of n=6-7 per group.


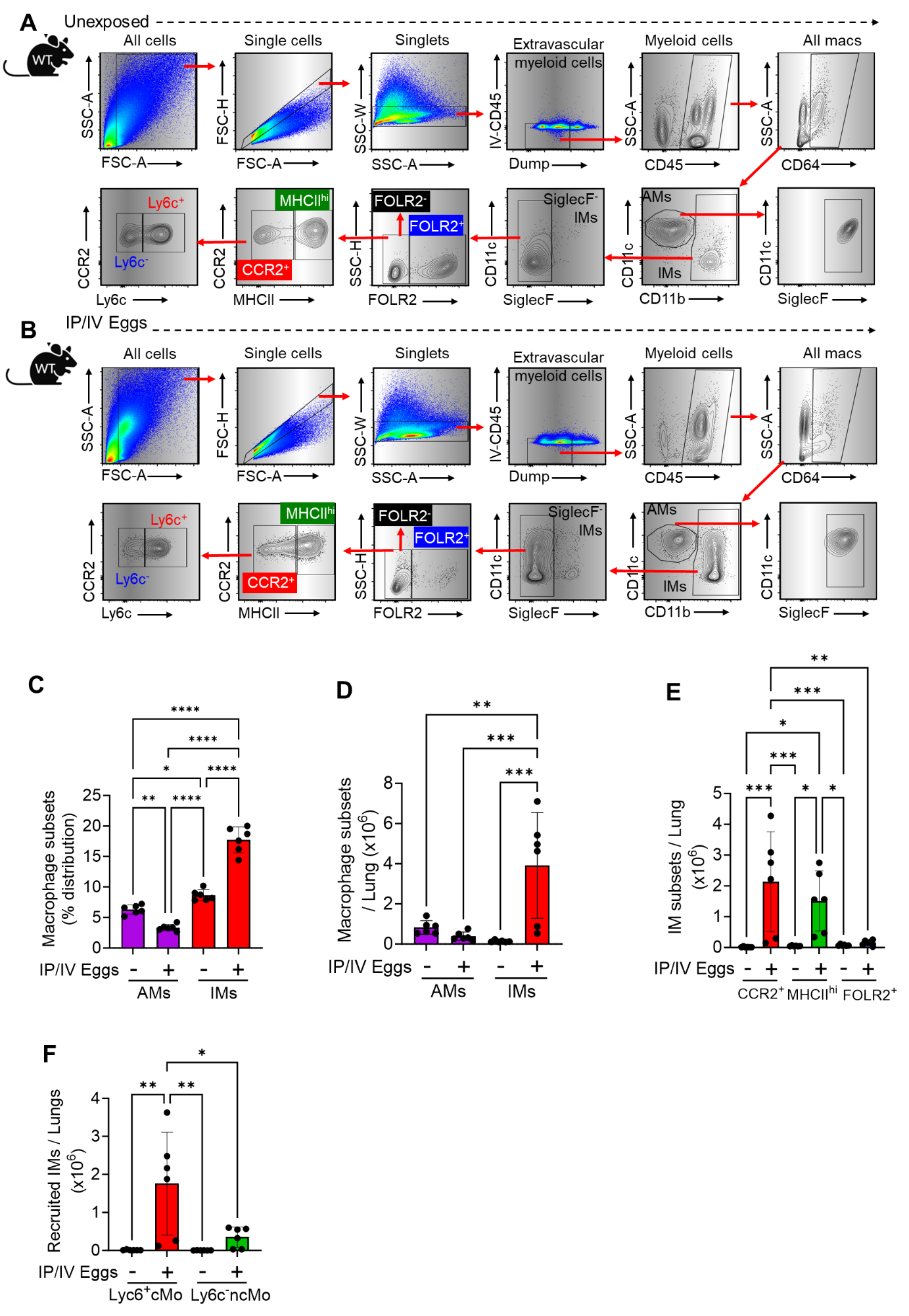


**Figure S2. Flow cytometry data on control and *Schistosoma* exposed wildtype mice with the addition of SiglecF and Ly6c gating**. Gating strategy to characterize IM subpopulations in (**A**) control and (**B**) *Schistosoma*-exposed wildtype mice. First singlets were gated negative for intravascular anti-CD45 and Lin-, and then gated extracellular CD45+ and then CD64+ to identify macrophages. The macrophages were divided into AMs and IMs based on CD11b and CD11c expression. The AMs and IMs were then further characterized by SiglecF expression, by gating the IMs additionally negative for SiglecF. These IMs were then subdivided by FOLR2 expression into FOLR2^+^ and FOLR2^-^ cells. The FOLR2^-^ population was further subdivided by CCR2 and MHCII expression as CCR2^+^ or MHCII^hi^ IMs. The Ly6c expression in the CCR2+ cells was interrogated to distinguish those with low expression as classical monocyte (cMo)-derived IMs versus those with high expression as nonclassical (ncMo)-derived IMs. Representative images of n=6-7 per group. Percentage and absolute number of (**C & D**) AMs and IMs, absolute number of (**E**) IM subpopulations, and (**F**) cMo versus ncMo phenotype in the CCR2+ subpopulation, by Ly6c expression status as in the gating strategy above, in unexposed and *Schistosoma*-exposed mice. N=6-7 per group. Abbreviations: AM: alveolar macrophage; IM: interstitial macrophage; IP: intraperitoneal; IV: intravenous. ANOVA with post-hoc Tukey test; P-value: **P*<0.05; ***P*<0.01; ****P*<0.001; *****P*<0.0001.

**
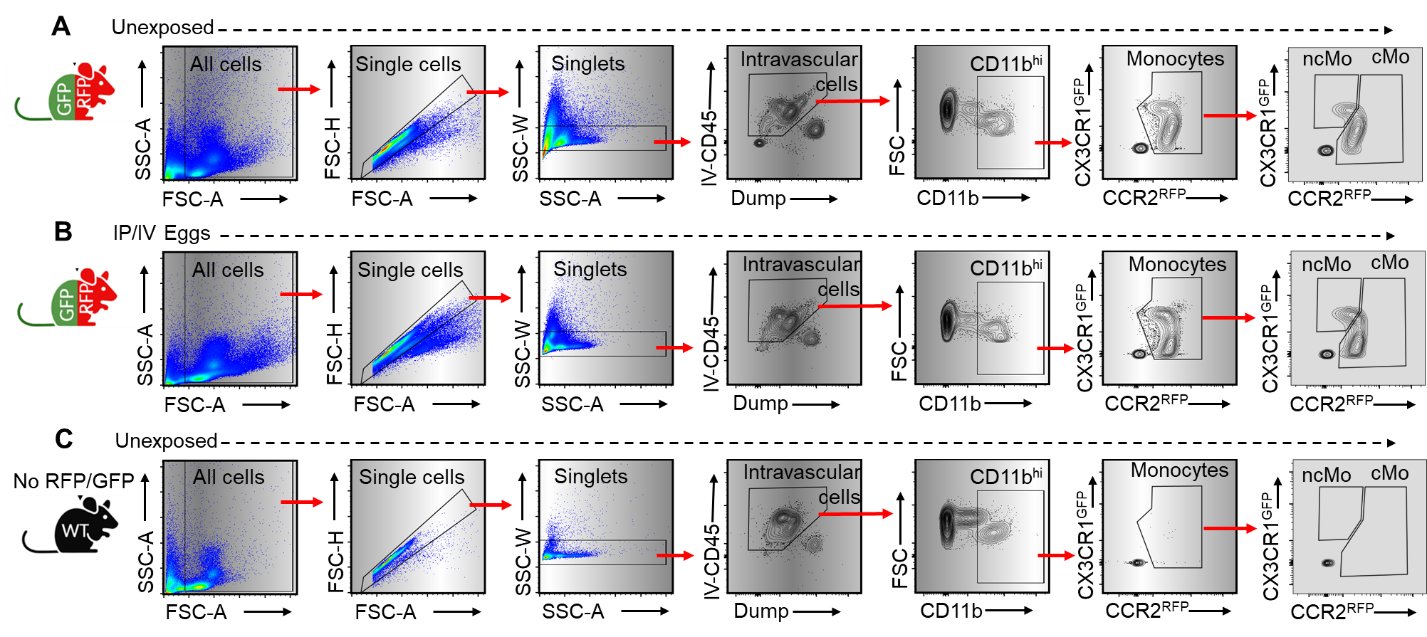
**

**Figure** **S3: Flow cytometry gating strategy for PBMCs to identify intravascular monocytes in unexposed and *Schistosoma*-exposed *Ccr2^RFP^Cx3cr1^gfp^* mice.** (**A-B**) Gating strategy in the double reporter mice to identify monocytes in PBMCs. Singlets were gated for Lin- and positive for intravenous CD45 to identify intravascular leukocytes, followed by CD11b^+^ gating, and then CCR2-RFP and CX3CR1-GFP expression. (**C**) PBMCs from wildtype mice served as controls for GFP and RFP gating. PBMC: peripheral blood mononuclear cell; GFP, green fluorescent protein; RFP, red fluorescent protein. These are representative images of n=6 per group.


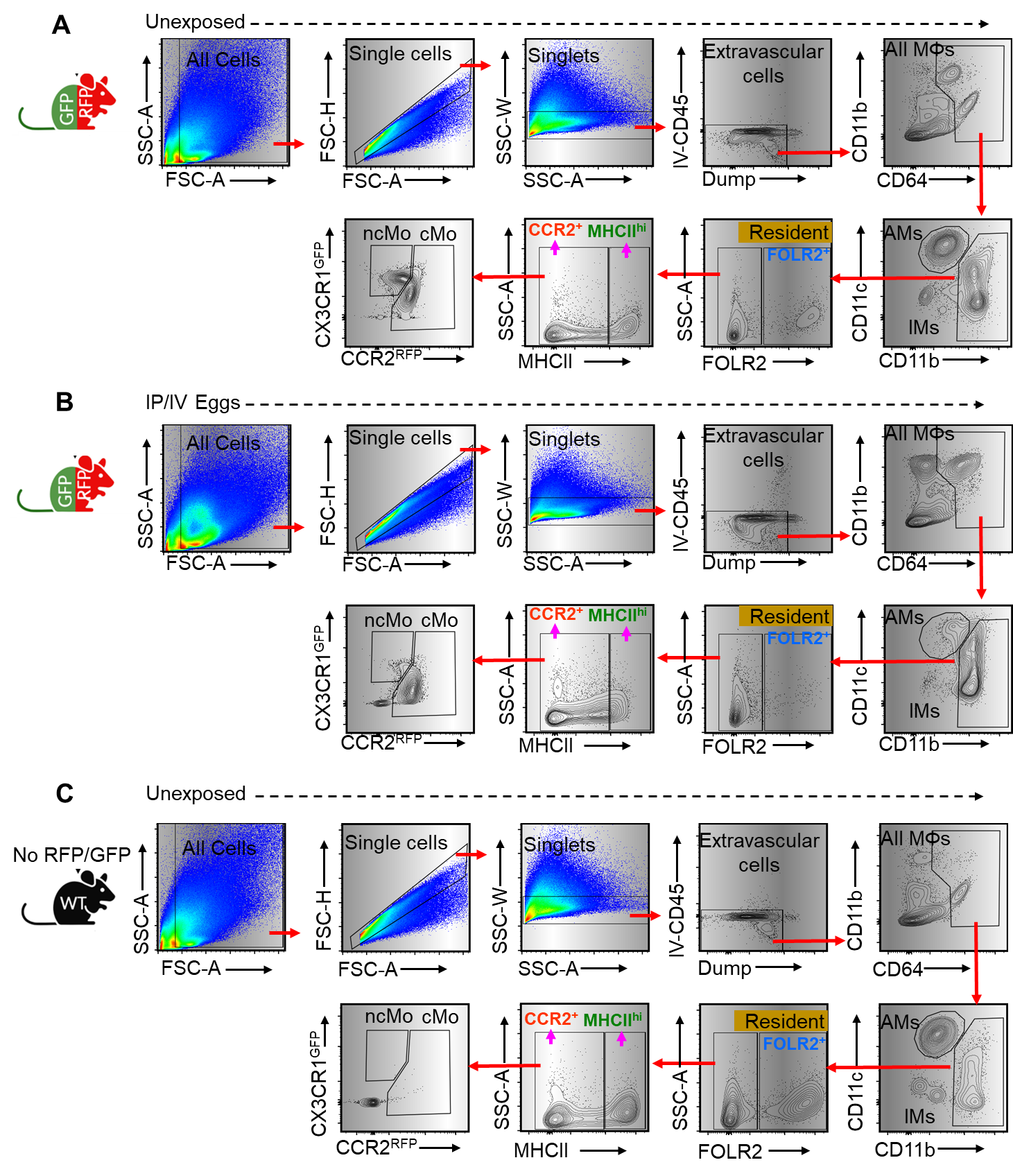
**Figure** **S4: Schematic representation of flow cytometry gating of to identify 3 pulmonary IM subpopulations in unexposed and *Schistosoma*-exposed *Ccr2^RFP^Cx3cr1^gfp^* mice**. Gating strategy in (**A**) control and (**B**) *Schistosoma*-exposed double reporter mice. Singlets were gated negative for intravascular anti-CD45, and Lin-, then CD64^+^ to identify macrophages, and then by CD11b and CD11c expression to identify AMs and IMs. The IMs were then characterized by FOLR2 expression and FOLR2+ and FOLR2- subsets were characterized. Further, FOLR2^-^ subsets were subdivided based on MHCII expression and MHCII^hi^ and CCR2^+^ subsets were identified. CCR2+ subsets. Based on the RFP and GFP expression, we identified CCR2+ IMs as monocytes derived GFP^lo^RFP^hi^ subpopulations. (**C**) Wildtype mice served as GFP and RFP controls for the reporter mice. IP, intraperitoneal; IV, intravenous; AM: alveolar macrophage; IM: interstitial macrophage; FOLR2, folate receptor 2, SSC-A, side scatter area; FSCA, forward scatter area. These are representative images of n=6 per group.


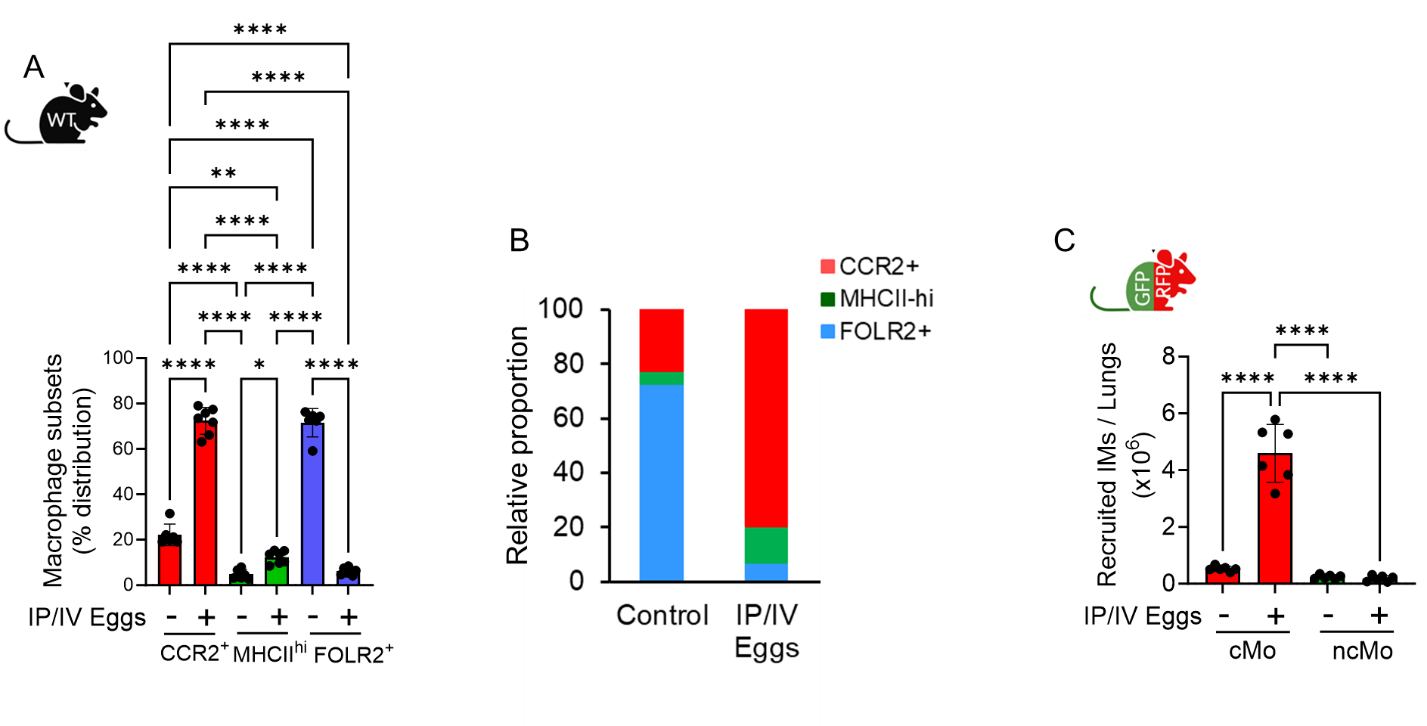


**Figure S5. Percentage and relative proportion of IM subpopulations.** (**A**) Percentage of IM subtypes out of all IMs and (**B**) the relative proportions of the 3 IM subpopulations following *Schistosoma* exposure in wildtype mice. (**C**) The RFP and GFP expression in the CCR2^+^ IMs was interrogated to distinguish those with GFP^lo^RFP^hi^ expression as classical monocyte (cMo)-derived IMs versus those with GFP^hi^RFP^lo^ expression as nonclassical (ncMo)-derived IMs. IP, intraperitoneal; IV, intravenous; ANOVA Tukey t-test **P*<0.05; *****P*<0.0001. N=6-7/group.


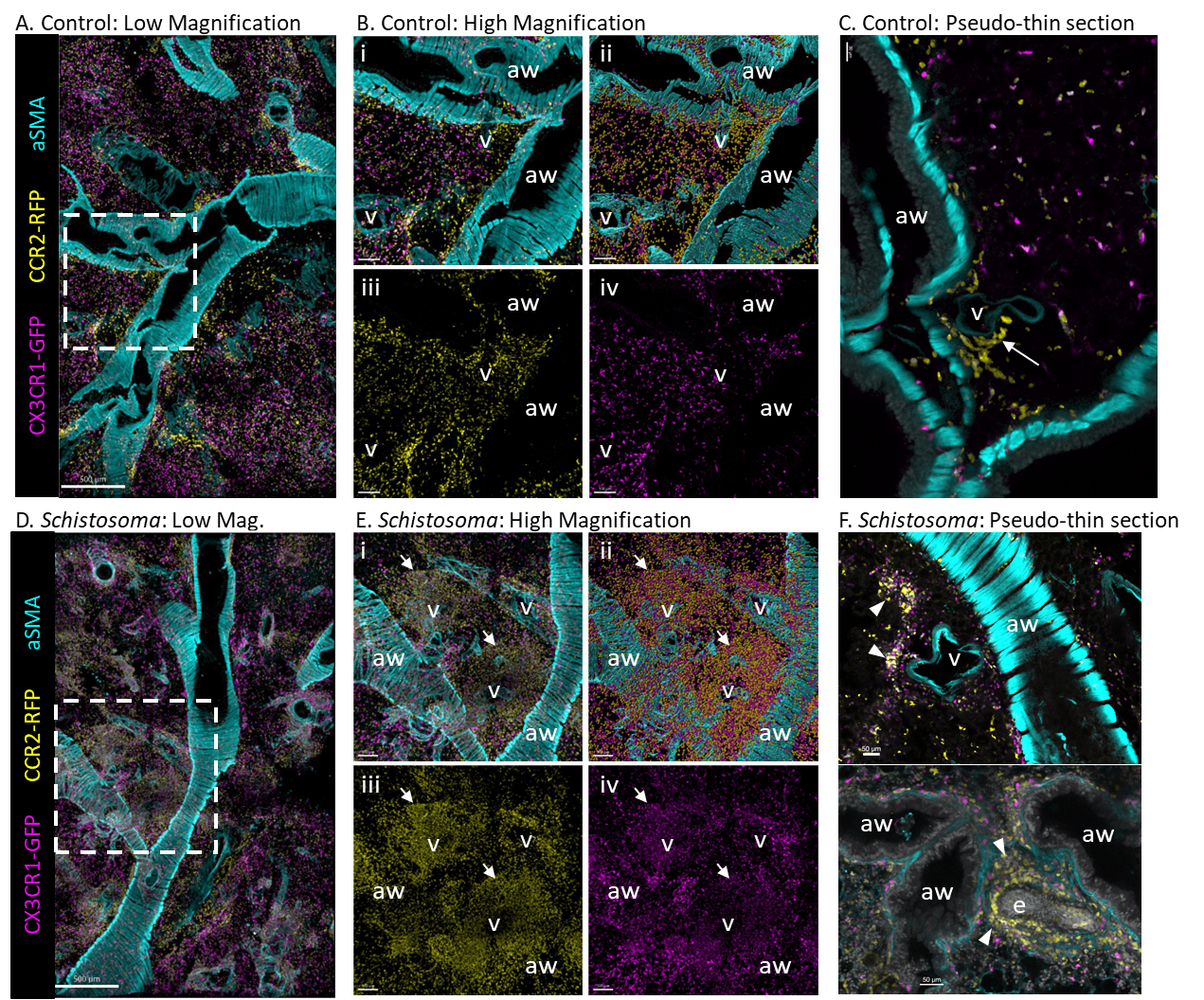


**Figure S6. Imaging of *Cx3cr1*^gfp^-*Ccr2*^rfp^ reporter mice reveals increased cell density in the perivascular region after *Schistosoma* exposure.** Otherwise unchallenged *Cx3cr1*^gfp^-*Ccr2*^rfp^ mice, imaged at (**A**) low magnification (scale bar 100µm) and (**B**) high magnification (scale bar 50µm; the dotted line in panel A). Panel (i) Composite image of all 3 wavelengths; (ii) Surfaced images, identifying individual cells; (iii) CCR2^RFP^ channel only; (iv) CX3CR1^GFP^ channel only; aw: airway; v: vessel. (**C**) Analysis of unchallenged mouse lung using pseudo-thin section imaging, identifying clusters of CCR2^RFP^ cells adjacent to vessels (indicated by long arrow). *Cx3cr1*^gfp^-*Ccr2*^rfp^ mice intraperitoneally (IP) sensitized and then intravenously (IV) challenged with *Schistosoma* eggs, imaged at (**D**) low magnification (scale bar 100µm) and (**E**) high magnification (scale bar 50µm; the dotted line in panel D). Panels (i)-(iv) are as in (B) above; short arrows indicate areas of increased infiltrates around the vessels. (**F**) Pseudo-thin sectioning identifies clusters of double-positive cells adjacent to vessels, indicated by arrowheads; e: *Schistosoma* egg within a vessel (scale bars 50µm).


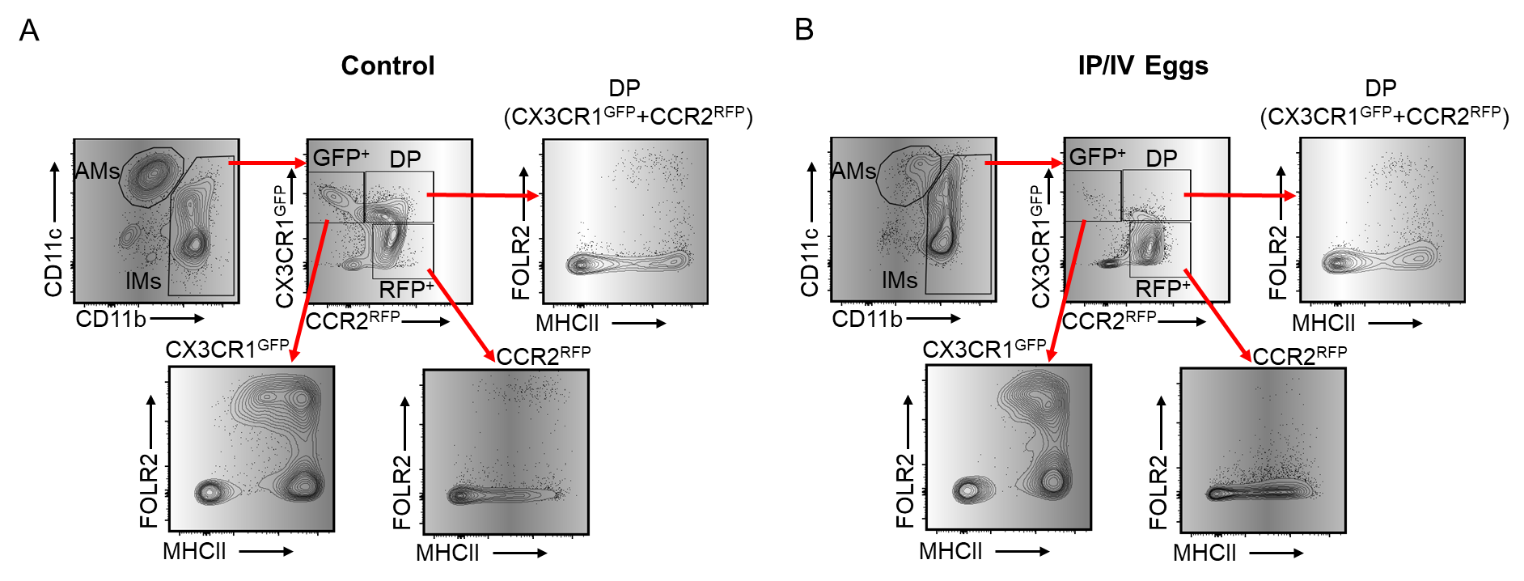


**Figure S7. Characterization of IM subpopulations based on CX3CR1^GFP^ and CCR2^RFP^ expression by flow cytometry in double reporter mice**. Representative gating of (**A**) control mice and (**B**) *Schistosoma* egg sensitized and challenged mice, based on GFP and RFP expression. GFP single positive (CX3CR1^GFP^) cells were identified as substantially FOLR2^+^, corresponding to FOLR2+ IMs, with some contribution of FOLR2^-^MHC2^hi^ and FOLR2^-^MHC2^lo^ IMs as well. RFP single positive (CCR2^RFP^) cells were characterized as FOLR2^-^, and a combination of MHC2^hi^ and MHC2^lo^ cells, corresponding to MHC2^hi^ and CCR2^+^ IMs. Cells marked by both GFP and RFP double-positive expression subsets (CX3CR1^GFP^ and CCR2^RFP^) were also characterized as FOLR2^-^ and a combination of MHC2^hi^ and MHC2^lo^ (ie, CCR2^+^) IMs.


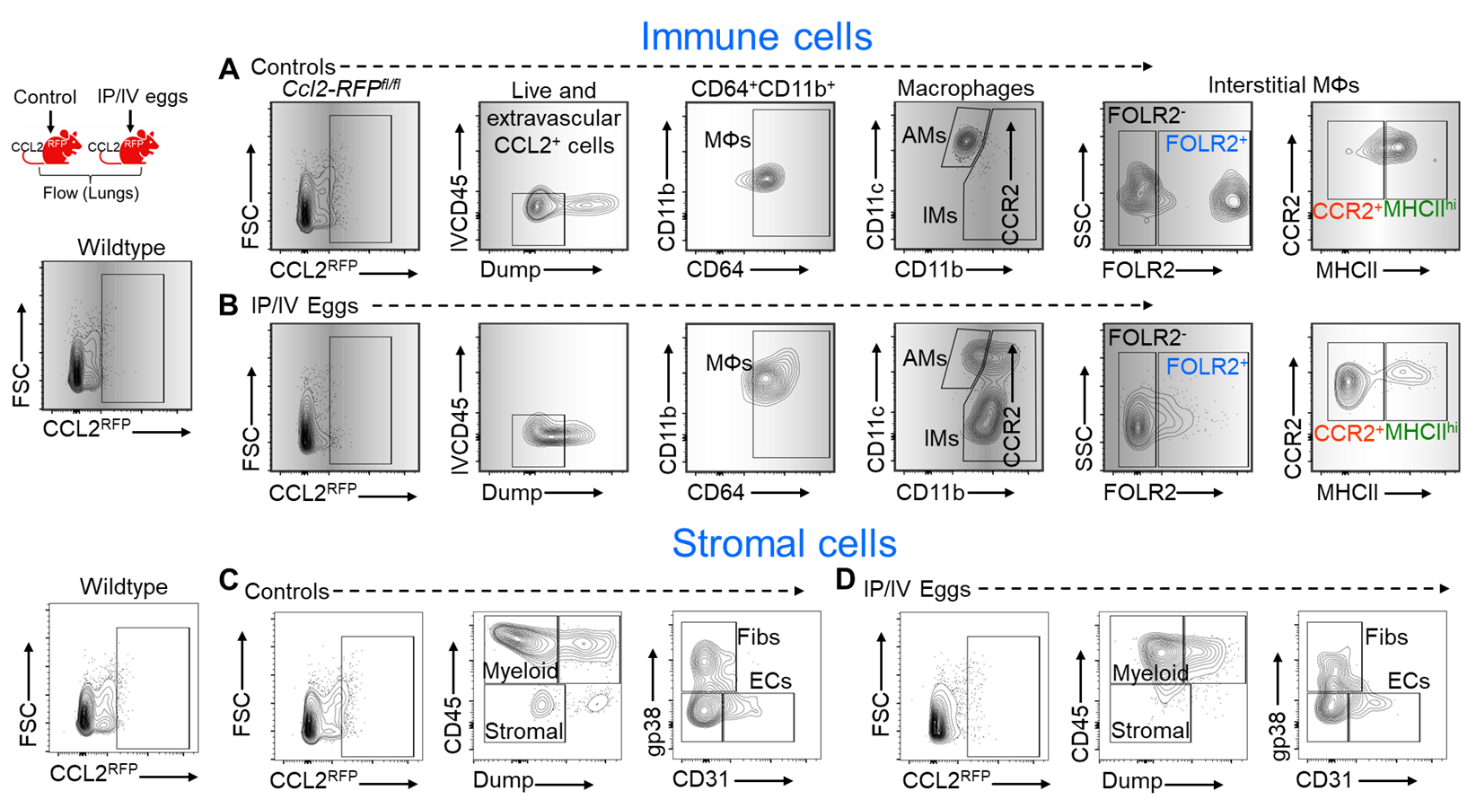


**Figure S8. Schematic representation of flow cytometry gating of control and *Schistosoma* exposed *Ccl2^RFP^* mice**. Gating strategy for flow cytometry in (**A**) unexposed and (**B**) *Schistosoma*-exposed *Ccl2^RFP^* mice. Singlets were gated for RFP^+^ cells, using a threshold identified by wildtype mice (left-most panel). The cells were then gated negative for intravascular CD45 and Lin-, and then positive for CD64 to identify macrophages, and differentiated into AMs and IMs by CD11b and CD11c expression. The IMs were further characterized into CCR2^+^, MHCII^hi^ and FOLR2^+^ subpopulations by first gating on FOLR2 expression to identify FOLR2^+^ IMs, and the FOLR2^-^ cells were then divided into CCR2^+^ and MHCII^hi^ IMs by CCR2 and MHCII expression. To identify RFP expression among stromal cells, (**C**) unexposed and (**D**) *Schistosoma*-exposed cells were gated RFP^+^ (using a threshold set by a wildtype mouse—left-most panel), negative for CD45, and then characterized by CD31 and gp38 expression as endothelial cells (ECs) or fibroblasts (Fibs). Representative images of n=6-7 per group.


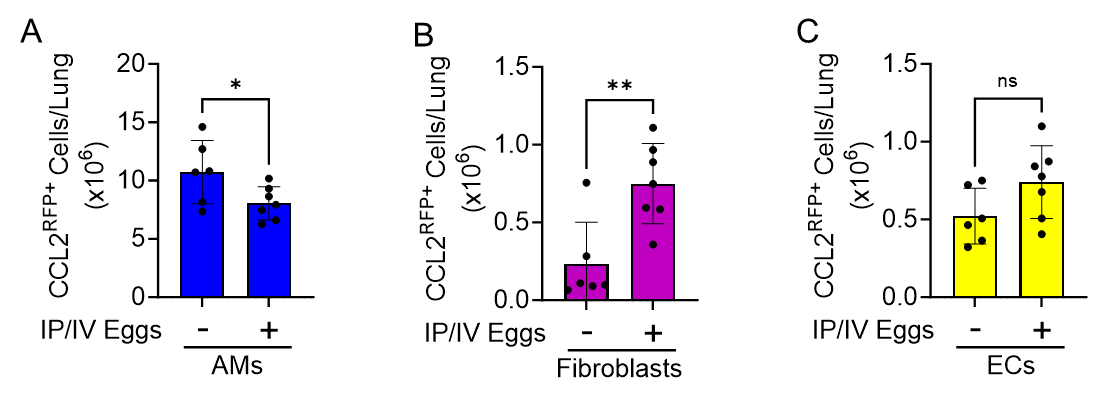


**Figure S9.** Absolute number of CCL2-RFP positive alveolar macrophages, fibroblasts and endothelial cells in *Ccl2^RFP^* mice, either control or 3 days after *Schistosoma* egg exposure. (**A**) Alveolar macrophages (AMs), (**B**) fibroblasts and (**C**) endothelial cells (ECs). n=6-7/group. T-test P values: **P*<0.05, ***P*<0.01.

**
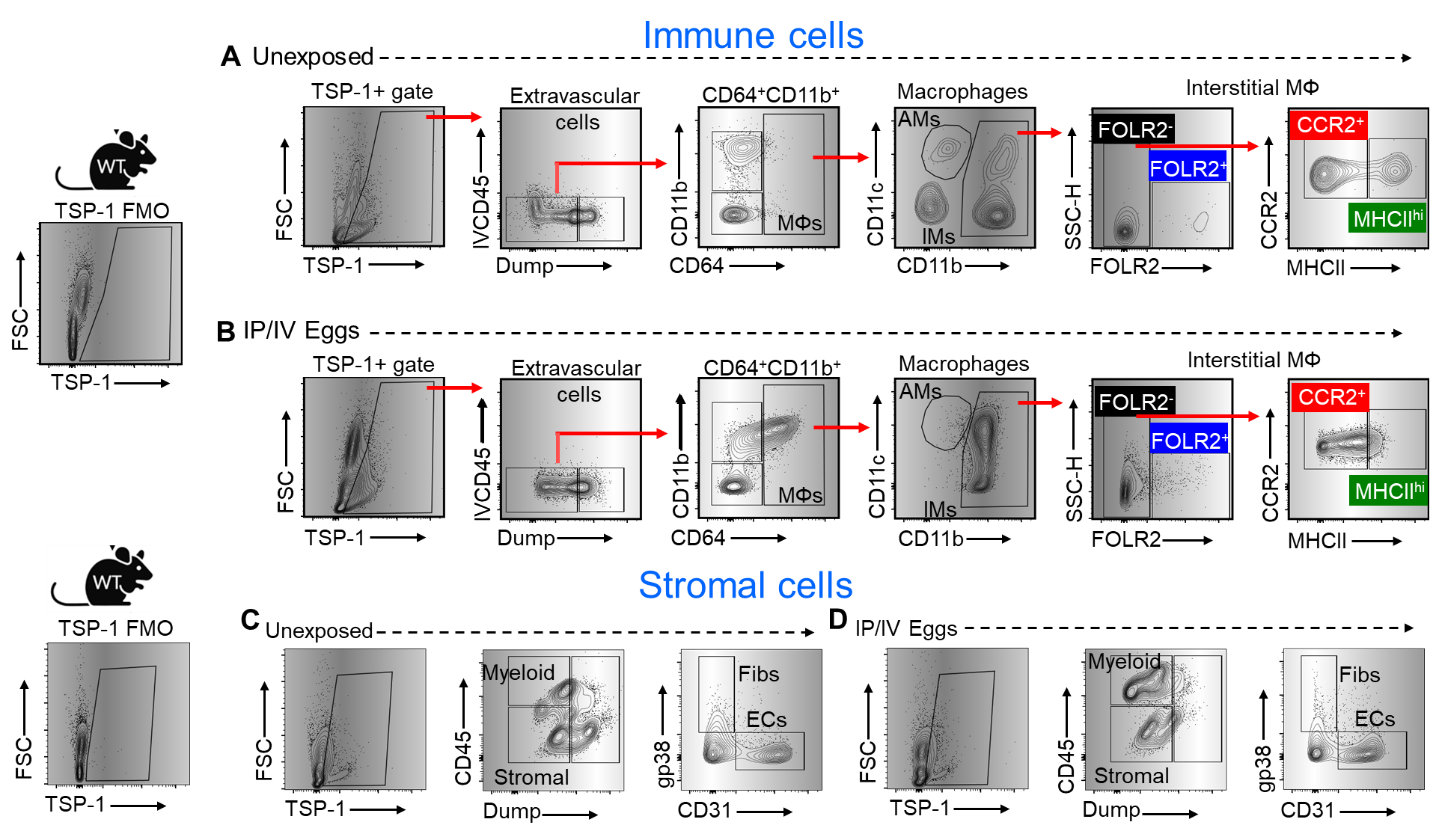
**

**Figure S10. Schematic representation of flow cytometry gating to identify intracellular TSP-1 expressing cells in unexposed and *Schistosoma*-exposed wildtype mice**. Gating strategy to characterize TSP-1 expression in immune cells in (**A**) control and (**B**) *Schistosoma*-exposed wildtype mice. Singlets were gated for TSP-1+ cells, using a threshold set by fluorescence minus one (FMO—see left-most panel). The cells were then gated negative for intravascular anti-CD45, and Lin-, and then CD64^+^ to identify macrophages, which were further subdivided into alveolar and interstitial macrophages by CD11b and CD11c expression. The IMs were further characterized into CCR2^+^, MHCII^hi^ and FOLR2^+^ subsets by first gating on FOLR2 to identify FOLR2^+^ IMs, and the FOLR2^-^ population into CCR2^+^ and MHCII^hi^ by CCR2 and MHCII expression. To identify TSP-1 expression among stromal cells, cells from (**C**) control and (**D**) *Schistosoma*-exposed mice were gated positive for TSP-1 using a threshold set by TSP-1 FMO—see left-most panel, negative for CD45, and then characterized by CD31 and gp38 expression as endothelial cells (ECs) or fibroblasts (Fibs). Representative images of n=6-7 per group.

**
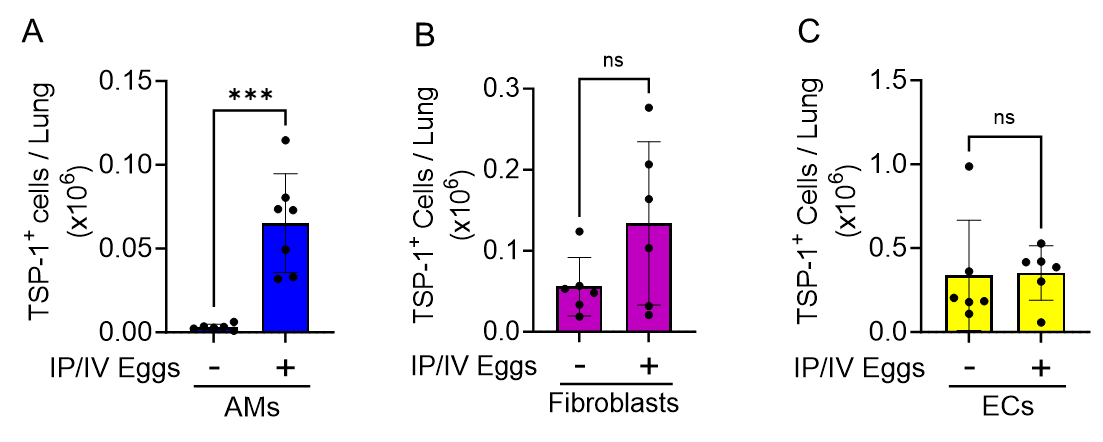
Figure S11. Absolute number of TSP-1 positive cells in alveolar macrophages, fibroblasts and endothelial cells in control and *Schistosoma*-exposed wildtype mice**. (**A**) Alveolar macrophages (AMs), (**B**) fibroblasts and (**C**) endothelial cells. n=6-7/group. T-test *P* values: **P*<0.05, ***P*<0.01, ns=nonsignificant.


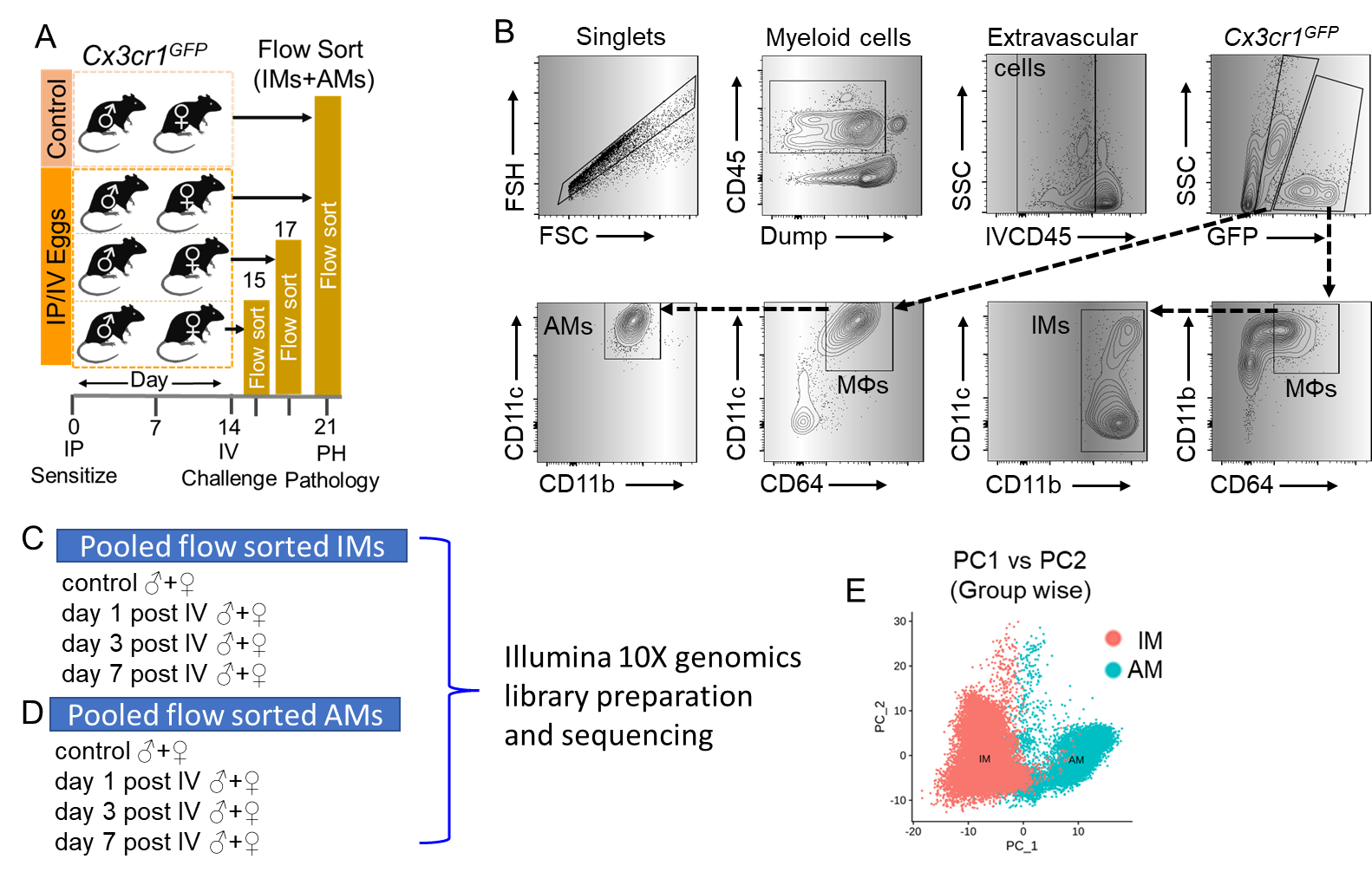


**Figure S12: Overview of the scRNAseq experiment to characterize IMs in unexposed and *Schistosoma*-exposed *Cx3cr1^GFP^* mice.** (**A**) Summary of the experimental design: mice were IP sensitized with *Schistosoma* eggs, and then 2 weeks later IV challenged with *Schistosoma* eggs. Sacrifice and flow sorting was done 1-, 3- and 7-days following IV eggs injection. Controls were *Cx3cr1^GFP^* unexposed mice. (**B**) Gating strategy for flow sorting. Singlets were gated CD45^+^ and Lin^-^ (excluding T cells, B cells, natural killer cells and neutrophils). Intravascular cells were then excluded by negative gating for intravenous anti-CD45. The cells were further gated based on GFP^hi^ expression, and then CD64^+^ and CD11b^+^ expression, whereas AMs were gated as GFP^lo^ CD64^+^ CD11c^+^. In each experimental group, the (**C**) IMs and (**D**) AMs were sorted from 1 male and 1 female mouse, mixed, and then used for library preparation and sequencing using the 10X Illumina platform. (**E**) Principal component (PC) analysis showed a clear separation of IMs and AMs populations.


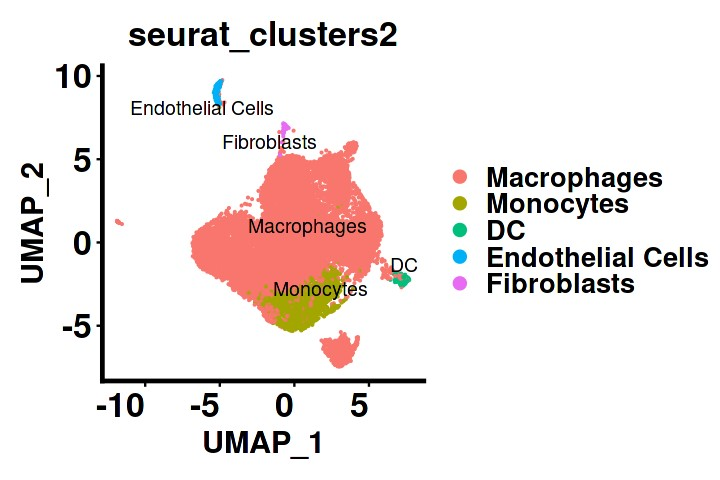


**Figure S13: ImmGen database categorization of scRNAseq clusters using Single R.** UMAP visualization of all cells that passed quality control, showing identification of different cell types, including macrophages, monocytes, endothelial cells, dendritic cells (DC), and fibroblasts.


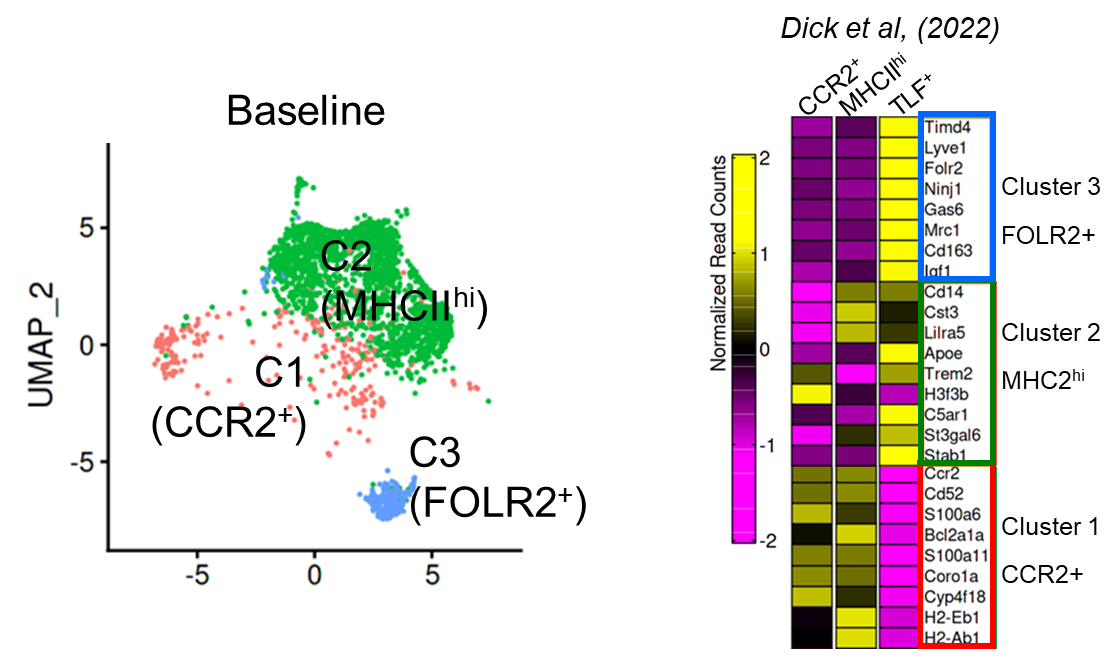


**Figure** **S14: Characterization of 3 IMs subpopulations in control animals by scRNAseq, compared to published gene lists.** Dick et al (15) identified top genes for each IM subpopulation, which identified as TLF^+^, MHCII^hi^, and CCR2^+^. This heatmap shows the expression of this gene list from Dick et al, in the 3 subsets characterized here, identified as FOLR2^+^, MHCII^hi^, and CCR2^+^.

**
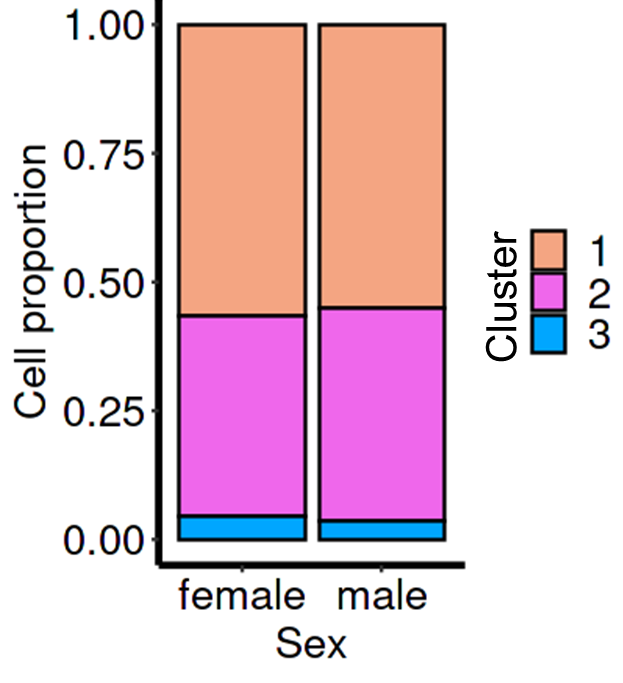
**

**Figure** **S15: Absence of sexual dimorphism of IM subpopulations.** Distribution of IM subpopulations across the 4 groups (control, and days 1, 3 and 7 following *Schistosoma* exposure), from male versus female mice.


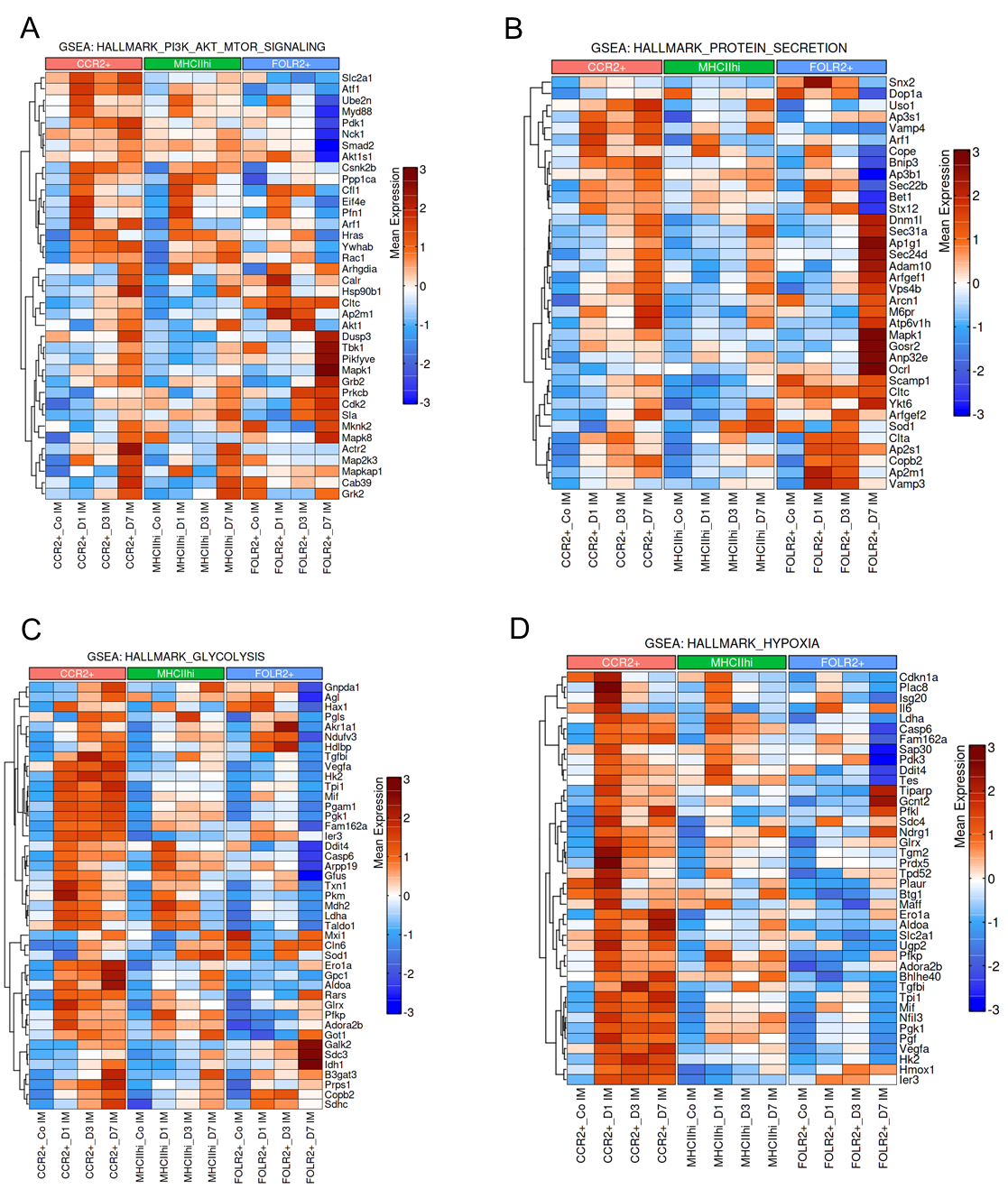


**Figure S16**: **Differential gene expression by IM subpopulation and timepoint of additional hallmark pathways.** Heatmap of genes associated with (**A**) PI3K-AKT-mTOR, (**B**) protein secretion, (**C**) glycolysis and (**D**) hypoxia response pathways using GSEA.


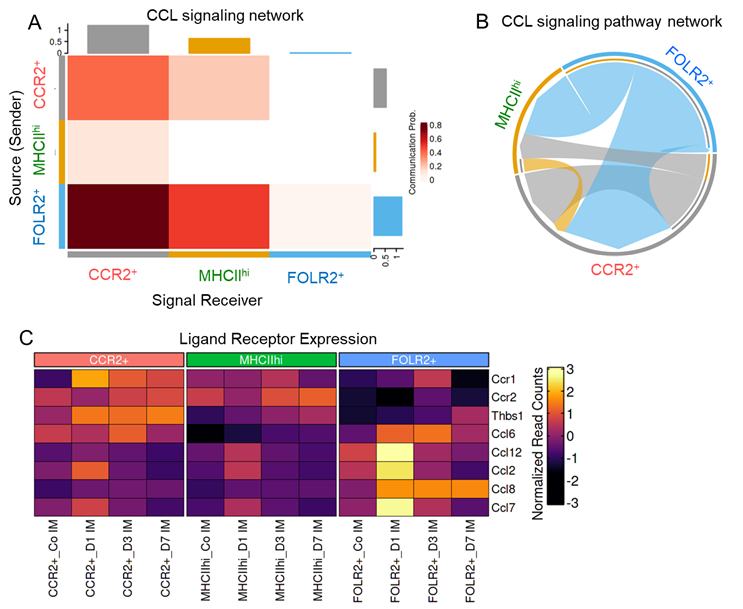


**Figure S17: Cell-to-Cell Communication.** (**A**) Matching of outgoing (ligand) and incoming (receptor) signaling patterns between IM subpopulations. (**B**) Directionality and degree of signaling between the IM subpopulations. (**C**) Heatmap of gene expression levels of CCRs and CCLs, and *Thbs1*.


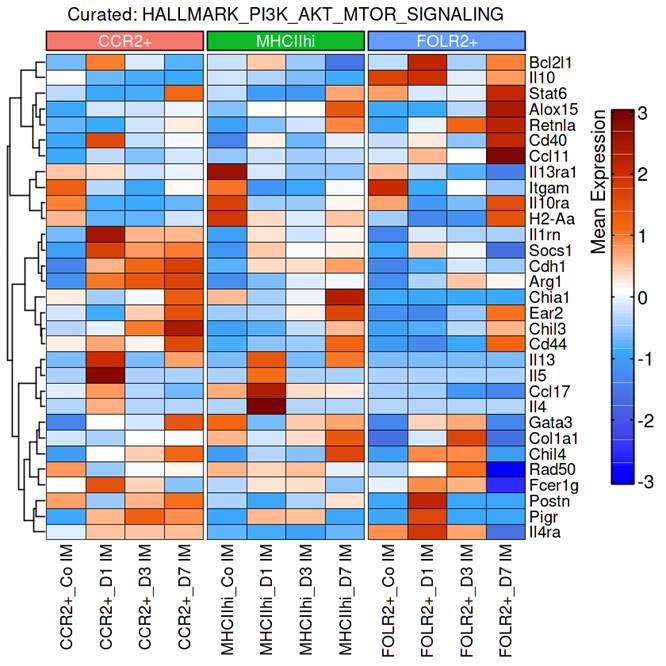


**Figure S18**: **Type 2 gene expression by IM subpopulations.**


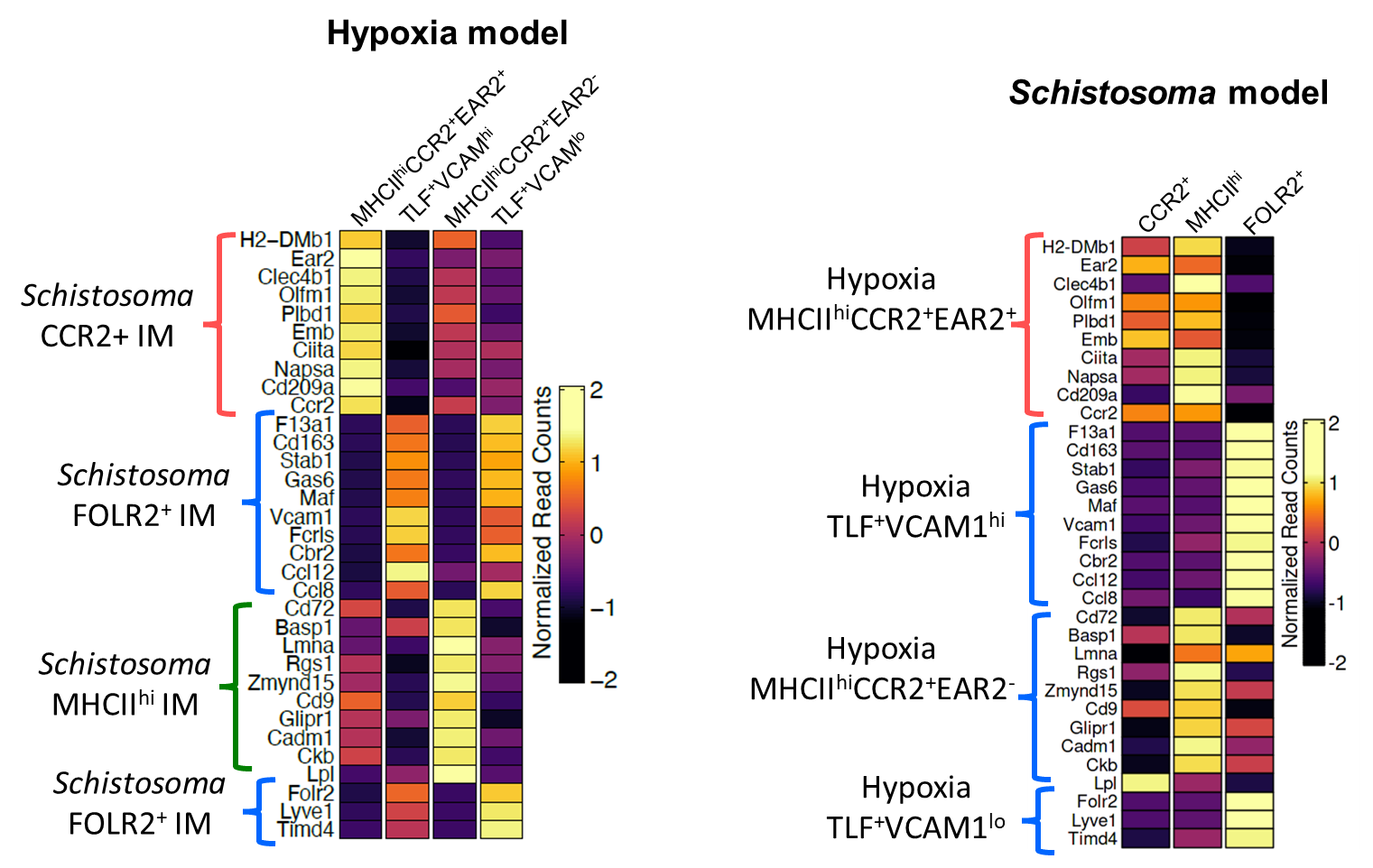


**Figure S19**: **Comparative analysis between hypoxia and *Schistosoma* model IM subtypes**. Top conserved markers of the hypoxia model within the three IM subsets characterized in the *Schistosoma* model across all timepoints.


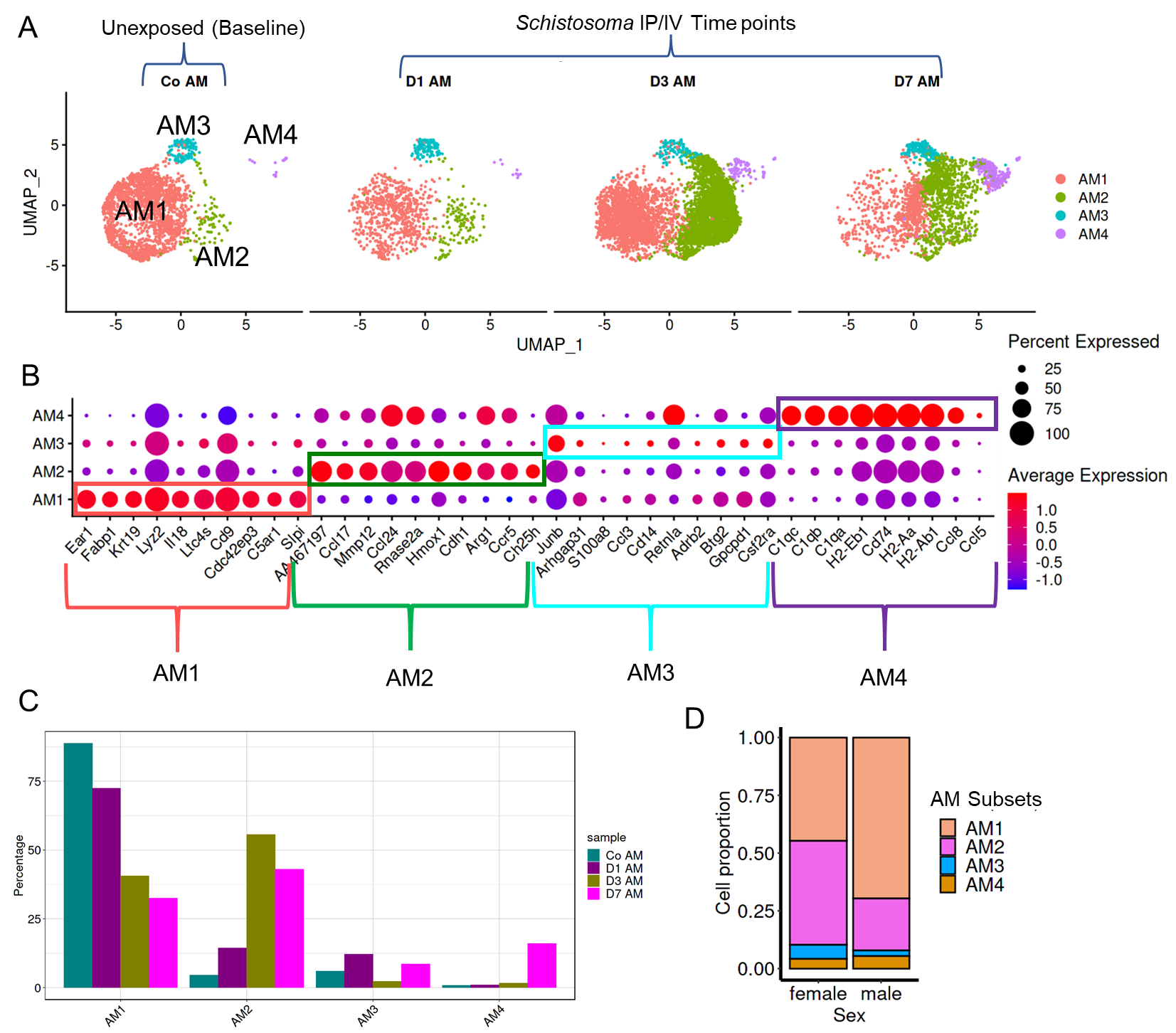


**Figure S20**: **Single cell RNAseq characterization of alveolar macrophages (AMs) in *Schistosoma*-induced inflammation.** (**A**) UMAP plot of 4 AM clusters in control mice, and at 1, 3 and 7 days after *Schistosoma* exposure. (**B**) Expression of key gene signatures at baseline, 1, 3 and 7 days after *Schistosoma* exposure in the 4 identified AM subpopulations. (**C**) Relative percentage of the 4 clusters at each timepoint. (**D**) Distribution of IM subpopulations across the 4 timepoints (control, and days 1, 3 and 7 following *Schistosoma* exposure), from male versus female mice.


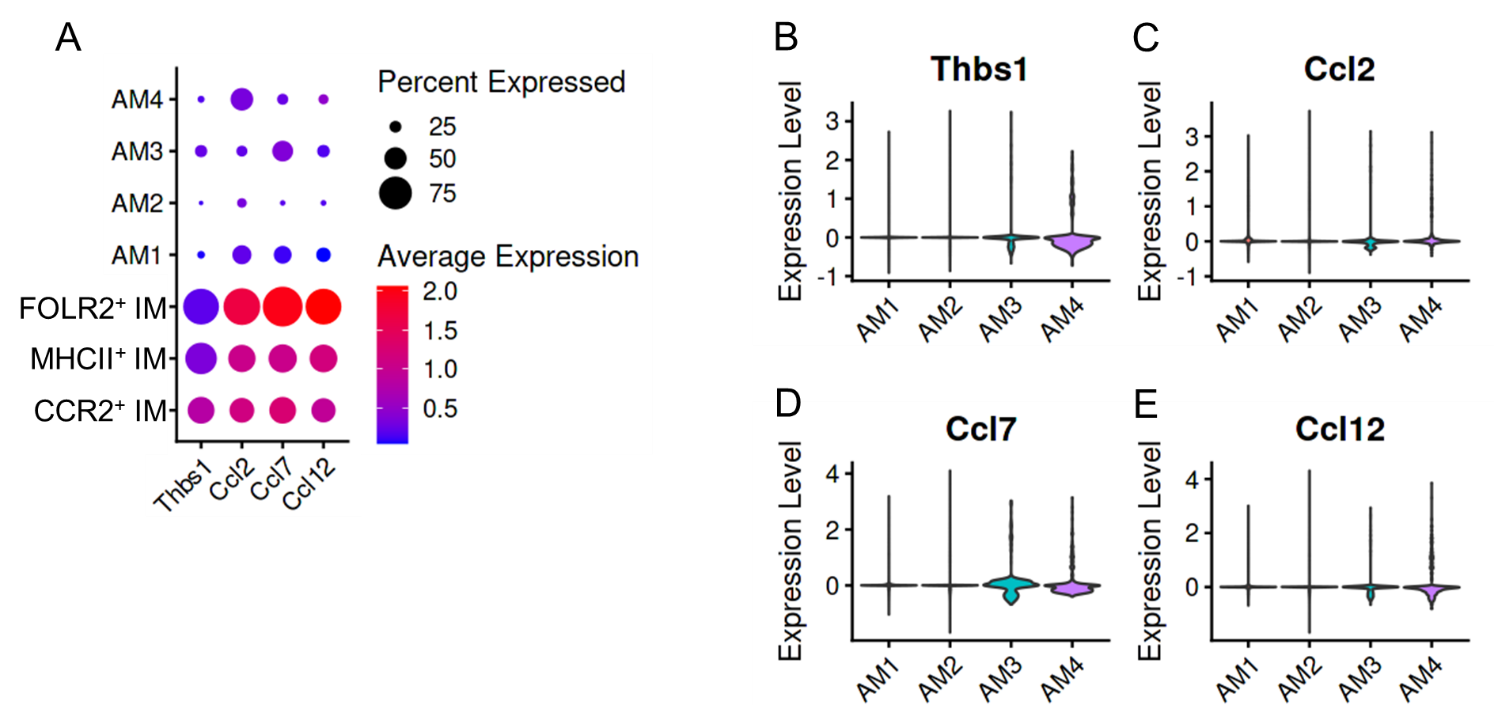


**Figure S21**: **TSP-1 and CCR2 ligand expression in AM subpopulations by scRNAseq**. (**A**) Cluster-based dot plot expression of Thbs1 (the gene encoding TSP-1) and CCR2 ligands Ccl2, Ccl5, and Ccl12 in the 4 AM subpopulations, as compared to the 3 IM subpopulations in *Schistosoma* PH (IM data as shown in **Figure 4**). (**B-E**) Violin plots showing the overall expression of *Thbs1, Ccl2*, *Ccl7* and *Ccl12* by the AM clusters.


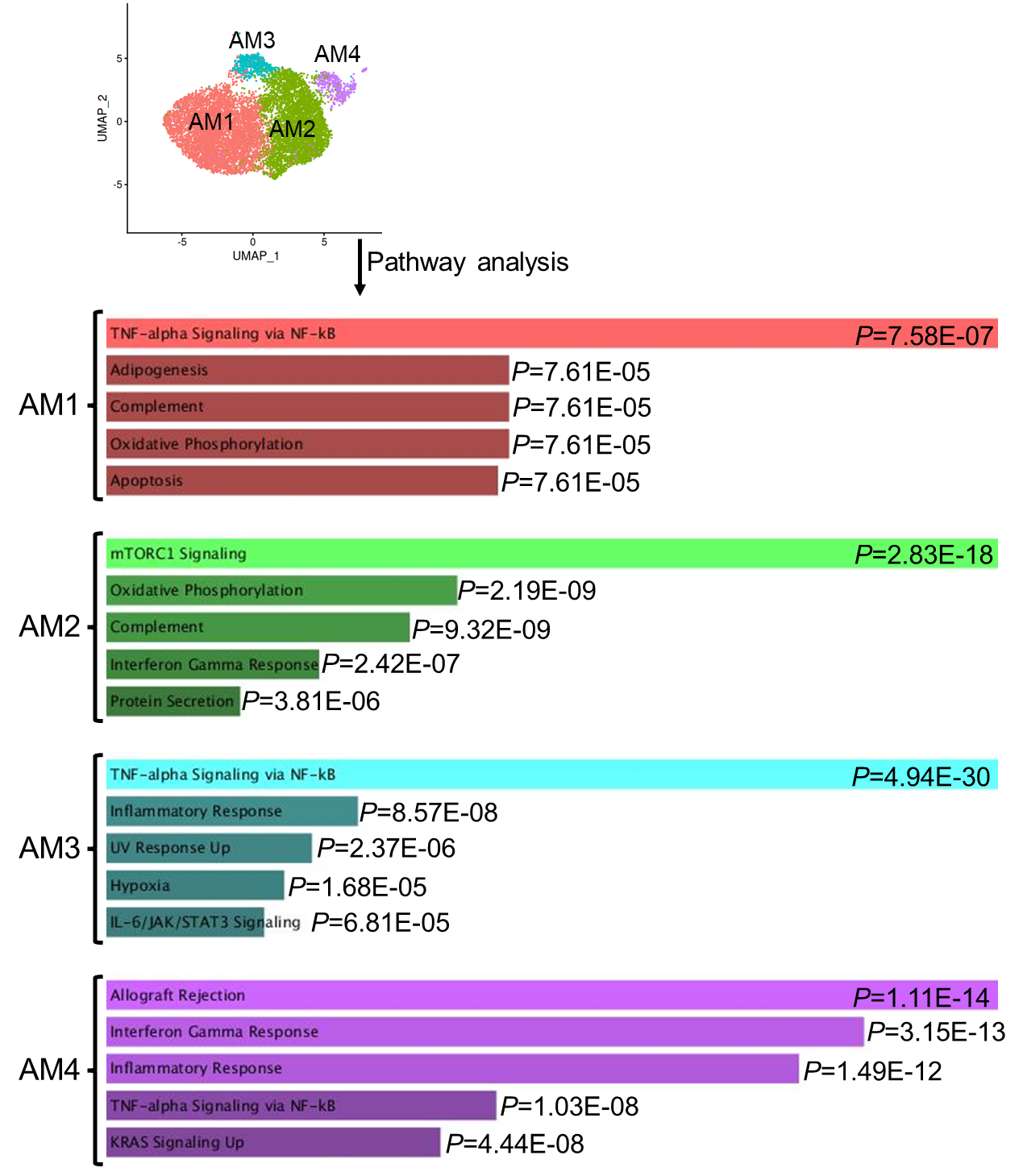


**Figure S22**: **Pathway analysis based on AMs clusters**: The bar charts identify the top five enriched pathways for each AM subpopulation utilizing the Hallmark database. The Enrichr program was utilized to conduct the pathway analysis. An adjusted statistical significance (*P*-value) is provided alongside each pathway.
